# Identification and Characterization of Contrasting Genotypes/Cultivars for Developing Heat Tolerance in Agricultural Crops: Current Status and Prospects

**DOI:** 10.3389/fpls.2020.587264

**Published:** 2020-10-22

**Authors:** Shikha Chaudhary, Poonam Devi, Anjali Bhardwaj, Uday Chand Jha, Kamal Dev Sharma, P. V. Vara Prasad, Kadambot H. M. Siddique, H. Bindumadhava, Shiv Kumar, Harsh Nayyar

**Affiliations:** ^1^Department of Botany, Panjab University, Chandigarh, India; ^2^Indian Institute of Pulses Research, Kanpur, India; ^3^Department of Agricultural Biotechnology, Chaudhary Sarwan Kumar Himachal Pradesh (CSK HP) Agricultural University, Palampur, India; ^4^Kansas State University, Manhattan, KS, United States; ^5^The UWA Institute of Agriculture, The University of Western Australia, Perth, WA, Australia; ^6^World Vegetable Center, International Crops Research Institute for the Semi-Arid Tropics (ICRISAT), Hyderabad, India; ^7^International Center for Agriculture Research in the Dry Areas (ICARDA), Rabat, Morocco

**Keywords:** heat-stress, crops, tolerance, agriculture, physiology

## Abstract

Rising global temperatures due to climate change are affecting crop performance in several regions of the world. High temperatures affect plants at various organizational levels, primarily accelerating phenology to limit biomass production and shortening reproductive phase to curtail flower and fruit numbers, thus resulting in severe yield losses. Besides, heat stress also disrupts normal growth, development, cellular metabolism, and gene expression, which alters shoot and root structures, branching patterns, leaf surface and orientation, and anatomical, structural, and functional aspects of leaves and flowers. The reproductive growth stage is crucial in plants’ life cycle, and susceptible to high temperatures, as reproductive processes are negatively impacted thus reducing crop yield. Genetic variation exists among genotypes of various crops to resist impacts of heat stress. Several screening studies have successfully phenotyped large populations of various crops to distinguish heat-tolerant and heat-sensitive genotypes using various traits, related to shoots (including leaves), flowers, fruits (pods, spikes, spikelets), and seeds (or grains), which have led to direct release of heat-tolerant cultivars in some cases (such as chickpea). In the present review, we discuss examples of contrasting genotypes for heat tolerance in different crops, involving many traits related to thermotolerance in leaves (membrane thermostability, photosynthetic efficiency, chlorophyll content, chlorophyll fluorescence, stomatal activity), flowers (pollen viability, pollen germination, fertilization, ovule viability), roots (architecture), biomolecules (antioxidants, osmolytes, phytohormones, heat-shock proteins, other stress proteins), and “omics” (phenomics, transcriptomics, genomics) approaches. The traits linked to heat tolerance can be introgressed into high yielding but heat-sensitive genotypes of crops to enhance their thermotolerance. Involving these traits will be useful for screening contrasting genotypes and would pave the way for characterizing the underlying molecular mechanisms, which could be valuable for engineering plants with enhanced thermotolerance. Wherever possible, we discussed breeding and biotechnological approaches for using these traits to develop heat-tolerant genotypes of various food crops.

## Introduction

**FIGURE 1:**
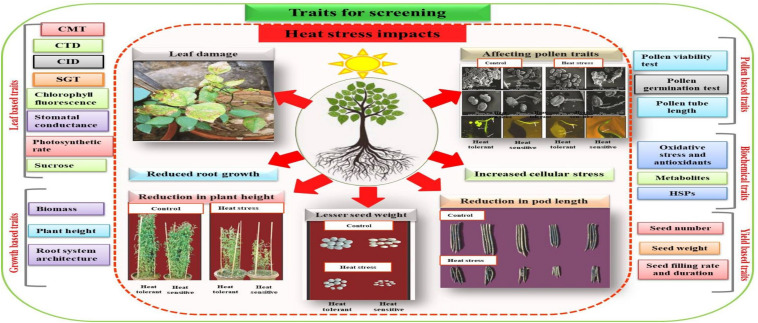
Screening traits for developing heat tolerant genotypes. Different traits based on leaf, growth, pollen grains biochemical and yield can be used for the selection of genotypes. Cell membrane thermostability (CMT), canopy temperature depression (CTD), carbon isotope discrimination (CID), stay green trait (SGT), chlorophyll fluorescence, stomatal conductance, photosynthetic rate and sucrose are the traits that can be assessed from leaves. However growth pattern such as plant biomass, plant height, and RSA of different genotypes can also be compared for selection of contrasting genotypes. Similarly, reproductive wellness of genotypes can be checked by using pollen based traits such as pollen viability test, pollen germination test and pollen tube length. The mitigation of cellular stress by genotypes can be compared by analysis of oxidative stress damage (production of free radicals) and production of antioxidants, metabolites and heat shock proteins (HSPs) whereas yield based traits such as seed number, seed weight, seed filling rate and duration can also be employed for selection purpose.

The Earth’s increasing average surface temperature due to climate change is proving to be stressful for all phases of plant growth and development, particularly in tropical and subtropical countries (Li B. et al., 2018). Among abiotic stresses, high temperature stress is a major factor disrupting plants’ performance (Wahid et al., 2007). Each plant species has its own maximum, optimum and minimum temperatures, known as cardinal temperatures. Temperatures below or above these thresholds causes stress (Wahid et al., 2007). Above optimum (high-temperatures) affect plant’s morphological, physiological, biochemical and molecular traits, which ultimately leads to poor growth and yields (Hasanuzzaman et al., 2013). The impact of high-temperature (heat) stress depends on intensity, timing, duration of stress and type of plant species (Wahid et al., 2007). Although all stages of plant development can be negatively impacted by heat stress, reproductive stages of crop are relatively more sensitive than vegetative stages (Prasad et al., 2008b, 2017). Heat stress during seed germination reduces germination percentage, seedling emergence, and radicle and plumule growth in germinated seedlings, resulting in abnormal seedlings with poor seedling vigor (Hasanuzzaman et al., 2013). At later vegetative stages, heat stress adversely affects photosynthesis, leaf area development leading to lower biomass production; whereas, stress during reproductive stages of development results in lower seed numbers and decrease seed size resulting in lower yields (Bita and Gerats, 2013; Prasad et al., 2017). Different crops and their genotypes vary in their heat sensitivity, the response is generally stage-and trait-specific, which can reveal mechanisms related to heat tolerance (Bita and Gerats, 2013; Prasad et al., 2017). Thus, genotypes having contrasting heat sensitivity have been identified in several crops (detailed below), that yielded vital information on various traits controlling heat tolerance (Figure 1).

## Impact of Heat Stress

Heat stress can have damaging effects (direct and indirect) on all stages of plant growth and development (Kaushal et al., 2016). Phenological stages differ in their sensitivity to heat stress, and vary between species and genotypes of same species. Various plant tissue injuries have been observed under heat stress, such as leaf and twig scorching, leaf, branch and stem chlorosis and necrosis, leaf senescence and abscission, root and shoot growth inhibition, flower drop, and fruitdamage, which consequently reduce plant productivity (Vollenweider and Günthardt-Goerg, 2005). Heat stress primarily affects the stability of plasma membranes, several proteins, cytoskeleton organization, and the efficiency of cell enzymatic reactions and creating metabolic disparity (Xu et al., 2006). Heat-stress-induced oxidative stress causes peroxidation of membrane lipids, proteins, and nucleic acids (Mittler et al., 2004). Due to reduced membrane stability, electrolyte leakage increases, which intensifies the membrane injuries (Wahid et al., 2007). Physiological processes, such as photosynthetic activity and sucrose metabolism, are highly sensitive to heat stress (Berry and Bjorkman, 1980). At the subcellular level, disruption of structural organization of thylakoids and loss of grana stacking in chloroplasts are the primary sites of heat injury (Sharkey, 2005), which lead to changes in electron transport to PSII (Lu and Zhang, 2000). Heat stress also damages PSII and inhibits its repair due to the generation of reactive oxygen species (ROS) (Allakhverdiev et al., 2008). Heat stress affects enzymes in Calvin cycle, including RuBisCo and RuBisCo activase (Camejo et al., 2005; HanumanthaRao et al., 2016; Bindumadhava et al., 2018), which hampers photosynthesis and photorespiration. Heat stress during reproductive stages adversely affects seed-set and yield in many food legumes, such as chickpea (*Cicer arietinum*) (Kaushal et al., 2013), mungbean (*Vigna radiata*) (Kaur et al., 2015; HanumanthaRao et al., 2016), peanut (*Arachis hypogaea*) (Prasad et al., 1999a,b) and lentil (*Lens culinaris*) (Bhandari et al., 2016) and cereals, such as wheat (Wahid et al., 2007; Prasad and Djanaguiraman, 2014), sorghum (*Sorghum bicolor*) (Prasad et al., 2015), barley (*Hordeum vulgare*) (Barnabás et al., 2008), and maize (*Zea mays*) (Kumar et al., 2012). During the reproductive stage, gametogenesis and fertilization are highly sensitive to heat stress, which impairs meiosis in both male and female organs, affects pollen germination and pollen tube growth, reduces ovule viability and ovule size, alters stigmatic and style positions, reduces stigma receptivity, disturbs fertilization processes, affects embryo fertilization, and impedes endosperm growth (Farooq et al., 2017; Prasad et al., 2017). Heat stress hastens the rate of grain filling, but reduces the duration of grain filling, as reported in wheat (Prasad et al., 2008a; Farooq et al., 2011), which may be due to direct effects of heat stress on the source–sink relationship that reduce photoassimilate supply to developing seeds (Calderini et al., 2006). The detrimental effects of heat stress can be alleviated by developing crop varieties with improved heat tolerance. The most popular approach used by many plant researchers has been screening a large population to identify contrasting genotypes for elucidating physiological, biochemical, and molecular mechanisms governing heat tolerance. Understanding such mechanisms will pave the way for improving crop genotypes under heat stress. Here, we discuss how heat stress impacts traits related to stress tolerance in contrasting genotypes of various crops to provide further clues for breeders and agronomists for improving the selection of heat-tolerant genotypes across crop species. Heat stress is often accompanied by drought stress; the impacts of heat stress are worsened in drought-stressed plants, which are manifested in various organizational changes in plants (Sehgal et al., 2017), hence, wherever possible, we have also included some examples where genotypes of crops have been screened against combined heat and drought stress situations.

## Mechanisms Governing Thermotolerance

Plants can endure two types of mechanisms to cope with heat stress: (1) basal thermotolerance (inherent ability of plant) and (2) acquired thermotolerance (induced by pre-exposure to higher but non-lethal temperatures) (Bokszczanin and Fragkostefanakis, 2013). Acquired thermotolerance has an important role to play in plant survival (Kotak et al., 2007). Hence, the heat stress response is a genetically controlled process that can be stimulated by mild or sub-lethal temperatures and further trigger the onset of heat-stress response in plants (Charng et al., 2006). The heat-stress response in plants is mainly conserved via cellular compartments and regulatory networks (Wahid et al., 2007). Plants have evolved various short-term acclimation mechanisms and long-term adaptations in response to heat stress. Short-term acclimation mechanisms include leaf orientation, transpirational cooling, and changes in membrane lipid composition (Wahid et al., 2007). For longer term adaptations, plants activate heat-stress tolerance mechanisms, such as heat stress sensing through various sensors and regulating downstream signal transduction pathways (e.g., lipidome, metabolome, transcriptome, and proteome) to modify gene expression to ensure survival (Sung et al., 2003; Bokszczanin and Fragkostefanakis, 2013; Dang et al., 2013).

Major adaptive mechanisms that induce thermotolerance in plants include amplified production of thermoprotectants, such as secondary metabolites, compatible solutes, ROS scavenging mechanisms, and heat-shock proteins (HSPs) (Nakamoto and Hiyama, 1999; Sakamoto and Murata, 2002; Wahid et al., 2007; Mittler et al., 2012). During severe heat stress, ROS generated as a byproduct of aerobic metabolism negatively affect cellular metabolism, such as peroxidation of lipid membranes and damage to nucleic acids and proteins (Bita and Gerats, 2013). Plants activate enzymatic and non-enzymatic ROS scavenging systems to defend this ROS production. The main ROS scavenging enzymes are superoxide dismutase (SOD), catalase (CAT), peroxidase (POX), ascorbate peroxidase (APX), and glutathione reductase (GR), and the non-enzymatic systems include ascorbic acid (ASC) and glutathione (GSH) (Suzuki et al., 2012). Elevated levels of these antioxidants are crucial for imparting thermotolerance in plants (Awasthi et al., 2015). Thus, to cope with heat stress, plants synthesize and accumulate more stress proteins, including HSPs, which are well-defined molecular chaperones involved in protein folding, and proper aggregation, translocation, and degradation under normal and stress conditions, and essential for sustaining cellular stability (Vierling, 1991). Five major families of HSPs/chaperones are HSP60, HSP70, HSP90, HSP100, and the small HSP (sHSP) family (Wang et al., 2004), which play an important role in the mitigation of heat-stress effects, especially protecting native proteins from denaturation. The accumulation of secondary metabolites, such as carotenoids, isoprenoids, and flavonoids, augments high-temperature stress tolerance by preventing peroxidase activity (Havaux, 1998; Loreto et al., 1998; Rivero et al., 2004). The accumulation of compatible solutes, *viz.* sugars (trehalose), sugar alcohols (polyols), proline, glycine betaine, tertiary, and quaternary ammonium compounds, also provides heat-stress tolerance in plants (Sakamoto and Murata, 2002). Due to their low molecular weight, these molecules can buffer the cellular redox potential under heat stress. Phytohormones, such as salicylic acid, abscisic acid, brassinosteroids, and polyamines, also play a significant role in providing thermotolerance to plants (Ahammed and Yu, 2016; Sharma et al., 2020).

## Growth-Based Parameters

Most studies on contrasting genotypes have measured biomass, plant height, and root growth, with significant variations identified in various crops subjected to heat stress, either in laboratory or field experiments, which has resulted in using these traits to quantify the impact of heat stress. Some examples of the impacts of heat stress on these traits are described below.

### Plant Height

Vegetative growth can be assessed as plant height to distinguish heat-stress tolerant genotypes (Debnath et al., 2016). In *Brassica juncea* L., high temperature (34°C) after the induction of flowering significantly affected plant height when grown in the field, declining by 18.9–30.5% (mean 22.3%), relative to the control. Genotypes BPR-538-10, NRCDR-2, RH-0216 had lower heat susceptibility, based on plant height, than genotypes RGN193, NPJ112 and SKM531 (Chauhan et al., 2009). Heat stress (>40°C) reduced plant height in 20 maize genotypes in the field (Debnath et al., 2016), with the most heat-tolerant genotype (DTPYC9F119) declining by 2.31% compared with a 72.2% reduction in the most heat-sensitive genotype LM13. Assessment of five potato cultivars (L1: 84.194.30; L2:86.61.26; L3: 87HW13.7, L4: DG81-68, and L5: Desiree) under controlled environment of combined heat (30 + 1°C) and drought stress (PEG 8000) for 21 days revealed severe effects all the cultivars on plant height, when both the stresses were together, except L2 and L3 (Handayani and Watanabe, 2020).

Seedling growth could be a potentially useful trait for early screening against heat stress. For instance, in some tropical parts of Africa, surface temperatures of tropical soils at planting time can exceed 50°C for hours to restrict the germination and seedling growth (Setimela et al., 2007). Hence, seedling heat tolerance is critical for adequate crop establishment in the semi-arid tropics. In a study carried out on sorghum (*Sorghum bicolor*) in Zimbabwe, seedling tolerance was estimated as heat tolerance index (HTI; defined as a ratio of resumed coleoptile growth after a controlled heat shock, compared to normal growth). Genetic parameters of HTI were assessed by crossing four sorghum lines having varying HTI, with three tester lines, and deriving F1, F2, F3, BC1, and BC11 families for generation means analysis. HTI was found to be highest (0.71) in Line IS20969 from Egypt, while an experimental line (290R), from the University of Nebraska, had the lowest at 0.51. The study revealed that additive and dominance effects contributed to coleoptile elongation under normal conditions, but only additive effects were significant in recovery growth. Epistatic effects were observed in both normal and heat-stressed environment. General combining ability (GCA) effects for HTI were highly marked in both conditions, but specific combining ability (SCA) effects were negligible. These results showed that it is achievable to enhance seedling heat tolerance and, thus, improve plant populations of sorghum in tropical areas where hot soil temperatures occur.

### Root System Architecture

The structure, spatial, and temporal configuration of the plant root system is called root system architecture (RSA) (de Dorlodot et al., 2007). The organization of primary and secondary roots is determined by RSA at the macroscale (Smith and De Smet, 2012). Root microstructure, such as fine root hairs, root tips and their interactions with soil and soil microorganisms responsible for water and mineral uptake, is determined by RSA at the microscale (Wu et al., 2018). Most resources are heterogeneously distributed in the soil, and the spatial and temporal distribution of roots determines the crop’s ability to exploit resources (Brussaard et al., 2007). Better understanding of RSA allows us to determine the impact of environmental conditions and management practices on crops, which can help to reduce the difference between genetic potential and actual average yields (Garnett et al., 2009; Judd et al., 2015; Ryan et al., 2016). RSA has a vital role in plant–soil–microbe interactions and resolves the crosstalk with beneficial soil microbes in the rhizosphere (Ryan et al., 2016).

Across fluctuating environments, RSA is an important characteristic for adaptability. Therefore, we can improve crop performance in terms of increased root traits, such as allocation, morphological, anatomical, or developmental plasticity (Sultan, 2000). There is a direct relationship between individual RSA plasticity and yield, which is related to more stable plant performance across changing environments in various species (Sadras, 2009; Niones et al., 2012, 2013). Root branching is important for improving the root surface area, enabling the plant to reach more distant reserves of water and nutrients and improve soil anchorage. In plants, heat stress generally reduces primary root length, lateral root density (number of lateral roots per unit primary root length) and angle of emergence of lateral roots from the primary root, but has little effect on average lateral root length (McMichael and Quisenberry, 1993; Nagel et al., 2009). Root growth has a lower optimal growing temperature than shoot growth and is thus more sensitive to rising temperatures (Huang and Gao, 2000; Xu and Huang, 2000). Plant heat tolerance is directly influenced by root morphological features. Among Kentucky bluegrass (*Poa pratensis)* cultivars, increased root number and root length contributed to variations in heat tolerance (Lehman and Engelke, 1993). Root phenotyping of 577 common bean (*Phaseolus vulgaris* L.) genotypes in variable environments of heat, drought, and nutrient deficiency revealed significant relationships between seed yield and seedling basal root number, seedling adventitious root abundance, and seedling taproot length. Under heat stress, adventitious root number had a positive relationship (13%) with seed yield. Mesoamerican genotypes of common bean yielded higher than Andean genotypes under heat stress (Strock et al., 2019). In canola, heat stress reduced stem diameter by 8.4%, cross-sectional area by 17.3%, and aboveground biomass by 11.5% in two genotypes; genotype 13C204 (heat-sensitive) had smaller stem diameter, cross-sectional area, root length, root surface area, root biomass, and root volume than Invigor 5440 (heat-tolerant) (Wu et al., 2017). In another study, heat stress reduced lateral root elongation (–38%), number of axile roots (–30%), elongation rate of primary root (–26%), root dry weight (–39%), leaf water potential (–59%) and leaf area (19%) in heat-sensitive maize lines K64R, Ac7643, and Ac7729TZSRW when the temperature increased from 28 to 37°C. The heat-tolerant lines H16, CML444, and SC-Malawi were least affected by high temperature (Trachsel et al., 2010). In maize, screening of 10 genotypes for combined heat and drought stress (40°C/60%) revealed DK 6789, NT 6621 as tolerant and genotypes FH 988 and FH 1137 as sensitive on the basis of root tolerance indices (Ayub et al., 2020).

### Biomass

Wheat seedlings grown for 7 days under normal conditions and then subjected to heat stress (42°C for 2 h) in a growth chamber revealed growth differences between genotypes (Gupta et al., 2013). In general, heat stress reduced growth (shoot/root dry weight and shoot/root length). Heat-tolerant genotypes (Raj-4037 and PBW590) retained more shoot and root length and dry weight than heat-sensitive genotypes (PBW502, DBW16, DBW17, WH1021, and PBW550) (Gupta et al., 2013). In a field experiment, heat stress (30/20°C) reduced wheat biomass, relative to optimum conditions (25/15°C), but heat-tolerant genotypes (MW-8, BW-4, and BW-3) maintained more biomass than heat-sensitive genotypes (MW-7, MW-6, and BW-5) (Rahman et al., 2009). Heat stress (>32/20°C) significantly reduced chickpea biomass by 22–30%, relative to those grown under normal temperatures; heat stress had a smaller effect on the biomass of heat-tolerant genotypes (ICC15614 and ICCV92944) than heat-sensitive genotypes (ICC5912, ICC10685) (Kaushal et al., 2013). Similarly, in a field experiment on alfalfa (*Medicago sativa*), heat stress (38/35°C) reduced plant biomass, relative to the control (25°C), more so in heat-sensitive Wl712 than heat-tolerant Bara310SC (Wassie et al., 2019). Field studies on lentil revealed that heat stress (>32/20°C) significantly reduced plant biomass, relative to the control (Sita et al., 2017a); genotypes IG3263, IG2507, IG3297, IG3312, IGG3327, IG3330, IG3546, IG3745, IG4258, and FLIP2009 retained more biomass (termed heat-tolerant) than genotypes IG2519, IG2802, IG2506, IG2849, IG2821, IG2878, IG3326, IG3290, IG3973, IG3964, IG4242, DPL15, DP315, IG4221, and IG3568 (termed heat-sensitive). Likewise, heat stress (>40/28°C) significantly reduced mungbean biomass (up to 76%), relative to the control (34/16°C) in the field, due to the inhibition of vegetative growth and acceleration in reproductive growth. Genotypes EC693357, EC693358, EC693369, Harsha, and ML 1299 produced more biomass under heat stress (heat-tolerant) than genotypes EC693363, EC693361, KPS1, EC693370, and IPM02-3 (heat-sensitive) (Sharma et al., 2016). A study on potato (*Solanum tuberosum*) revealed that warmer temperatures (31/29°C) severely affected plant biomass in two genotypes—Norchip (heat-tolerant) and Up-to-date (heat-sensitive)—grown in controlled environment chambers (Lafta and Lorenzen, 1995). Both genotypes had similar total dry mass under controlled conditions (19/17°C), but heat stress (31/29°C) reduced total dry mass by up to 44% in Norchip and 72% in Up-to-date. Leaf, stem, shoot, and tuber dry and fresh weights followed the same trend under high temperature in both genotypes. At Niger, West Africa (ICRISAT Sahelian Centre research farm (13° 29≪ N, 2° 10≫ E; 221 m above sea level), field experiments were performed to evaluate heat tolerance of groundnut (*Arachis hypogaea* L.) using physiological traits identified in a yield model [crop growth rate (C), reproductive duration (Dr) and partitioning (p)]. After screening 625 diverse groundnut genotypes, under irrigation during the hottest months (February to May), 16 contrasting genotypes, selected on the basis of combination of high pod yield and partitioning coefficient, revealed that crop growth rate was a powerful factor affecting pod yield. Pod yield of most genotypes decreased by more than 50% because of heat stress (40°C) at the time of flowering and pod formation. The findings showed that estimates of p would be vital as a dependable selection criterion, compared to yield, for identification of heat tolerant genotypes. The breeders should explore strategies to maximize the crop growth rate and partitioning in genotypes growing under warm environments (Ntare et al., 2001). Under combined heat and drought (36/26°C without irrigation) stresses, Biomass was used as a trait for evaluation of 3 tomato cultivars (Arvento and two heat tolerant; LA1994 and LA2093) (Zhou et al., 2017) resulting in identification of “Areventro” cultivar as more tolerant than “LA1994” and “LA2093” genotypes (Zhou et al., 2017).

## Leaf-Based Traits

Heat stress causes serious leaf injuries, yellowing of leaves (chlorosis), tissue death (necrosis), especially tips and margins, wilting, and drying, resulting in severe loss of functionality (Wahid et al., 2007). Various traits have been used to assess heat damage, with genotypes contrasting for heat tolerance identified based on these traits.

### Tissue Damage

Tissue damage can be assessed by measuring membrane damage based on electrolyte leakage, which is a reliable indicator of heat sensitivity in several crop species. The primary target of environmental stress is the cell membrane (Chen et al., 2014; Sita et al., 2017b). Heat stress loosens chemical bonds within the molecules of biological membranes by accelerating the kinetic energy and movement of molecules across membranes, which results in membrane fluidity by protein denaturation or increased unsaturated fatty acids (Savchenko et al., 2002). Under high temperature, protein denaturation, increased membrane fluidity, and enzyme inactivation decreases protein synthesis and degradation, and alters membrane integrity (Howarth, 2005). The tertiary and quaternary structure of membrane proteins changes with heat stress and enhances membrane permeability, as evident from increased ionic leakage, which is an indicator of decreased cell membrane thermostability (CMT) (Wahid et al., 2007). Damage to leaf membranes occurs due to the direct effects of high temperature, photo-oxidation of chlorophyll pigments, impaired electron flow, inhibition of carbon fixation, and water loss from leaves. Damage to membranes impairs photo-assimilate production in leaves (Prasad et al., 2017). Under high temperature, the relationship between CMT and crop yield varies from plant to plant. CMT has been used as an indirect measure of heat stress tolerance in plant species, such as sorghum (Sullivan, 1997; Marcum, 1998), soybean (Martineau et al., 1979), potato and tomato (Chen et al., 1982), sorghum, wheat (Blum et al., 2001), cotton (Ashraf et al., 1994; Cottee et al., 2010), lentil (Sita et al., 2017a), chickpea (Kaushal et al., 2013), mungbean (Sharma et al., 2016), and barley (Wahid and Shabbir, 2005). Abro et al. (2015) identified several heat-tolerant cotton genotypes with high membrane thermostability at 50°C in the laboratory environment, which could be used for breeding purposes to develop heat-tolerant genotypes. During the late developmental phase of plants, membrane stability tends to decrease (Ahmad and Prasad, 2011). For breeding purposes, significant variations in membrane thermostability among genotypes could be used to improve selection (Hemantaranjan et al., 2014).

In wheat grown under high temperatures (45°C for 2 h), genotypes (Raj4037, PBW373) with high CMT (58.20, 55.43) during grain filling performed better than those (Raj4083, DBW16, PBW550) with low membrane thermostability (48.15, 50, 51.96). Under controlled conditions, membrane thermostability was maximum in WH1021 (64.13) and minimum in DBW16 (51.11) (Gupta et al., 2013). Similarly, CMT was markedly higher in heat-tolerant (56.83%) than heat-sensitive (31.43%) wheat genotypes during grain filling. Based on CMT, Bala and Sikder (2017) identified heat-tolerant wheat genotypes BAW-1143, BARI Gom-25, BARI Gom-26, and Prodip. At the seedling stage in wheat, CMT had a positive correlation with grain yield, grain weight (Saadalla et al., 1990), and biomass (Blum et al., 2001), indicating the effectiveness of this trait for assessing heat tolerance. In rice at 40°C, thermostability was closely related to crop yield potential (Maavimani and Saraswathi, 2014). In a comparative study of rice and maize grown under controlled high temperatures (40/35°C and 45/40°C), the rice genotypes (PR116, PR118) had greater electrolyte leakage (27.4–40.2%) than the maize genotypes (PMH1, PMH2) (19.2–26.2%) (Kumar et al., 2012). Similarly, among three rice cultivars, F60 and F733 were more heat-susceptible than F473 when grown at 40°C, with greater electrolyte leakage (20 and 15%) (Sanchez-Reinoso et al., 2014). Likewise, Yadav et al. (2014) used CMT as an effective screening parameters for selecting heat tolerant lines in Pearl millet. From the same study, the authors also identified H77/29-2 × CVJ-2-5-3-1-3 hybrid as heat tolerance based on seedling thermotolerance index. Under combined stresses (drought-42–45% of irrigated conditions) and heat (> 32/20°C), the drought tolerant chickpea genotypes were found to tolerate the two stresses more effectively than heat tolerant genotypes. For instance, genotypes ICC1356 (drought-tolerant) showed less damage to membranes than genotype ICC3776 (drought-sensitive), when subjected to both the stresses (Awasthi et al., 2017).

In legumes, a few studies have identified heat-tolerant and heat-sensitive genotypes. Based on the membrane stability test, chickpea was most sensitive to heat stress, relative to other legumes such as pigeon pea, groundnut, and soybean (Devasirvatham et al., 2012). Contrasting chickpea genotypes exposed to high temperatures (40/30°C and 45/35°C) varied markedly, with heat-tolerant genotypes (ICCV07110, ICCV92944) showing less membrane damage (22.6, 20.6%) than heat-sensitive genotypes (ICC14183, ICC5912) (30.4, 33.3%) (Kumar et al., 2013). A similar test conducted at 37/27°C reported up to 25% electrolyte leakage in chickpea seedlings (Pareek et al., 2019). A heat-tolerant genotype (ICC1205) had low electrolyte leakage (13–14%), indicating better cell membrane integrity. Screening of cowpea genotypes exposed to heat stress also revealed less leaf electrolyte leakage (35.8–36.7%) in heat-tolerant genotypes (H36, H8-9, DLS99) during flowering and pod set than heat-susceptible genotypes (CB5, CB3, DLS127) (66.2–79%) (Ismail and Hall, 1999). In lentil, heat tolerance was related to less membrane damage (<20%) in heat-tolerant genotypes (IG2507, IG3263, IG3745, IG4258, and FLIP2009) than heat-sensitive genotypes (IG2821, IG2849, IG4242, IG3973, IG3964) (> 30%) at 38/28°C and 40/30°C in a controlled environment (Sita et al., 2017a). In another study, lentil genotypes (Ranjan, Moitree, 14-4-1, IC201710, and IC208329) were reported as heat-tolerant based on cell membrane stability under field and growth chamber studies at 34°C (Choudhury et al., 2012). Barghi et al. (2013) reported the highest CMT in genotype Qazvin (98.13%) and regarded it as heat-tolerant, whereas genotype B4400 (33.19%) had the lowest CMT (heat-sensitive). Under high temperature (38/35°C), screening of 15 *Medicago* cultivars for CMT identified Bara310SC and WL712 as heat-tolerant (24.07%) and heat-sensitive (53.2%) cultivars, respectively, having minimum and maximum electrolyte leakage, respectively (Wassie et al., 2019).

Cotton displays heat sensitivity at various growth stages. Cotton genotypes grown in a controlled environment under optimal conditions (35/21 ± 2°C) for 30 days and then exposed to high temperature (46/30 ± 2°C) at the reproductive stage, by gradually increasing temperature by 2°C per day, were screened for CMT—cultivars FH-900, MNH-552, CRIS-19, and Karishma emerged as relatively heat-tolerant (thermostable) and FH-634, CIM-448, HR109-RT, and CIM-443 as heat-susceptible (Rahman et al., 2004). In a similar study at > 32°C, cotton genotypes B557 and NIAB-78 showed minimum electrolyte leakage (<40%) and were regarded as tolerant compared to genotypes MNH-554, FH682 and FH900 which showed maximum electrolyte leakage (>50%) (Rana et al., 2011). Abro et al. (2015) reported cotton varieties NIA-80, NIA-81, NIA-83, NIA-84, NIA-M-30, NIA-M31, NIA-HM-48, NIA-HM-327, NIA-H-32, NIA-HM-2-1, NIA-Bt1, NIA-Bt2, NIA-Perkh, CRIS-342, CRIS-134, and NIAB-111 and check variety Sadori as heat-tolerant using CMT as a screening parameter in both heat-stressed (44°C) and non-stressed (32°C) temperature regimes. Other similar studies where cotton genotypes were differentiated by CMT into heat-tolerant and heat-sensitive were conducted by Karademir et al. (2012); 15 genotypes; > 40°C) and Singh K. et al. (2018); 37 genotypes; > 40°C).

Likewise, in cucumber, contrasting genotypes were identified based on membrane stability under heat stress (40/32°C)—L3466 and Desi cucumber as heat-tolerant and Suyo Long and Poinsett as heat-sensitive (Ali et al., 2019). In tomato, 2 h exposure to high temperature (45°C) altered CMT more in heat-sensitive variety Campbell-28 (> 45%) than heat-tolerant variety Nagcarlang (<20%) (Camejo et al., 2005). In another study on 44 tomato lines, exposure to 44°C for 4 h after 1 week of vegetative stage increased electrolyte leakage in heat-sensitive genotypes (32.92 μmhos/cm) more than heat-tolerant genotypes (22.2 μmhos/cm) (Hameed et al., 2015). Similar studies have screened tomato genotypes for heat tolerance using membrane thermostability (Sangu et al., 2015; Alsamir et al., 2017). Thus, CMT is an effective trait for identifying stable and heat-tolerant genotypes.

### Canopy Temperature Depression

At the whole crop level, leaf temperatures decrease below air temperature when water evaporates. Canopy temperature depression (CTD)—the difference between air temperature (T_a_) and canopy temperature (T_c_)—acts as an indirect measure of transpiration (Reynolds et al., 2001) and plant water status (Araus et al., 2003). A positive CTD value is observed when the canopy is cooler than the air (CTD = T_a_–T_c_) (Balota et al., 2008). CTD is a heritable trait that can be measured on cloudless days with an infrared thermometer (Reynolds et al., 1998). Plants transpire through open stomata to maintain canopy temperature in a metabolically comfortable range. Under stress, plants close their stomata for some period, which increases the canopy temperature (Kashiwagi et al., 2008). Canopy temperature is affected by soil water status, wind, evapotranspiration, cloudiness, conduction systems, plant metabolism, air temperature, relative humidity, and continuous radiation (Reynolds et al., 2001). To assess heat tolerance, many traits can be used as selection criteria, but, CTD is considered to be best as a single reading integrates scores of leaves (Reynolds et al., 1994, 1998; Fischer et al., 1998). Yield potential and the metabolic fitness of crop plants under specific environmental conditions are determined by CTD (Kumari et al., 2013). A study on barley revealed a strong link between epicuticular leaf wax QTL and CTD, and that wax load influences plant canopy temperature (Awika et al., 2017). Based on phenotypic variation, CTD can act as a desirable criterion for heat-tolerant genotype selection (Mason and Singh, 2014). CTD is a mechanism of heat escape and has a strong genetic correlation with yield (Reynolds et al., 2001). Heat-tolerant genotypes of wheat had higher CTD than heat-sensitive genotypes, indicating their greater ability to maintain a cooler canopy environment (Gare et al., 2018). In another study, the CTD value in wheat was correlated with heat resilience (Pradhan et al., 2012). In 102 durum wheat genotypes tested under late-sown conditions, CTD had a strong positive correlation with days to maturity (Gautam et al., 2015), confirming that CTD is an effective selection criterion in plant breeding (Seema et al., 2014). Leaf area having more greenness and CTD are strongly interrelated in wheat and with grain yield, grain-filling duration, and biomass (Kumari et al., 2013). Stay-green genotypes have high CTD values due to transpirational cooling, resulting in lower canopy temperatures (Reynolds et al., 1994; Fischer et al., 1998). In stay-green lines, low CTD values delayed senescence (Kumari et al., 2013). Leaf width in wheat had a high correlation with canopy temperature under heat stress (Mohammadi et al., 2012). In durum wheat, CTD had a positive correlation with biological yield and spike number/m^2^ at first spikelet emergence and 50% inflorescence stages. At three growth stages (first spikelet emergence, 50% inflorescence, and completion of anthesis), harvest index had a negative correlation with CTD (Bahar et al., 2008). Screening of Indian and CIMMYT wheat germplasm for the stay-green trait and CTD revealed higher CTD values in the stay-green genotypes due to transpirational cooling and lower canopy temperatures (Kumari et al., 2013). In wheat (*Triticum aestivum*), heat stress reduced CTD by 39.7% at the grain-filling stage (Joshi et al., 2016). Timely sown wheat had higher CTD than late-sown wheat (Saxena et al., 2016), with genotypes HD2932, HD2864, HD3095, HI8703, and HUW234 identified as heat-tolerant due to their higher net photosynthesis, relative water content, membrane stability index and CTD than the other tested genotypes (Saxena et al., 2016). Additional management factors, such as the use of farmyard manure and NPK, improved physiological traits (light interception, CTD, and flag leaf chlorophyll content) in wheat (Badaruddin et al., 1999). In seven rice varieties, CTD was closely related to stomatal conductance and leaf photosynthetic rate (Takai et al., 2010). Rice varieties Takanari and TUAT1-5-6a had lower leaf temperatures and higher stomatal conductance and leaf photosynthetic rates than the other varieties tested under cloudy conditions. Infrared thermography, as a simple method of evaluating varietal differences in stomatal conductance via CTD, is feasible even under cloudy conditions. In chickpea, water potential, osmotic pressure, relative leaf water content, and seed yield had a negative correlation with CTD (Sharma D. K. et al., 2015). Heat-tolerant chickpea genotypes ICCVs 95311, 98902, 07109, and 92944 had higher CTD than sensitive genotypes ICCVs 07116, 07117, and 14592, which had negative CTD values (Devasirvatham et al., 2015). In mungbean, CTD had a significant positive correlation with seed yield, and a negative correlation with root traits, such as lateral branch number and dry root weight (Raina et al., 2019). Greater pod number and pod to node ratio was associated with CTD in pea (Tafesse et al., 2019). In cotton, the involvement of CTD in heat tolerance was indicated (Cornish et al., 1991), with additive, dominance, and epistatic components involved in its inheritance (Khan et al., 2014). In another study on cotton, crop development stage had no effect on CTD, which was significantly correlated with seed yield (Karademir et al., 2018). Canopy temperature in cotton increased under combined heat and drought stress treatment (>36°C and 35% irrigation) (Carmo-Silva et al., 2012), as compared to control. Low canopy temperature was noticed in cotton cultivar Pima S-6 (S6), which was reported as tolerant, unlike high canopy temperature in Monseratt Sea Island (MS), termed as sensitive, under combined stress.

### Stomatal Conductance

Under heat stress, regulating the transpirational mechanisms is a possible strategy for selecting heat-tolerant varieties (Condon et al., 2007). As leaves open their stomata, the rate of gaseous exchange may create differences in stomatal behavior that can be recorded by a leaf porometer (Chandra et al., 2017; Priya et al., 2018). Fully opened stomata increase the diffusion of CO_2_ and, at the same time, increase transpiration and photosynthetic efficiency in wheat (Condon et al., 2007). Consequently, stomatal regulation is an important factor that governs plant growth and survival. Therefore, stomatal conductance (*g*_S_) is a useful trait for determining photosynthetic and transpiration rates. Stomatal conductance increases with rising temperature (Urban et al., 2017). Crawford et al. (2012) suggested that plants acclimatize to high temperatures by evaporating more water, thereby keeping their canopies cool despite the presence of fewer stomata. Similarly, semi-dwarf spring wheat cultivars had strong positive correlations between *g*_S_ and photosynthetic rate, cooler canopies and yield (Fischer et al., 1998). Heat-tolerant advanced cotton lines (e.g., Pima S-6) developed by Cornish et al. (1991) had higher stomatal conductance and photosynthetic rates under heat stress, which was possibly due to cooling effect of plants through stomata. The stomatal conductance of 50 cotton genotypes was measured under high temperature (45–50°C/20–30°C day/night) in a glasshouse, and identified five heat-tolerant genotypes (NIAB-111/2, BH-160, MNH-554, N-313, BH-163, Mutant-94) (Khan et al., 2008). Similarly, 41 wheat lines of different origin were screened for higher *g*_S_, which was associated with heat tolerance (36/30°C for 1 week) (Sharma K. D. et al., 2015). Heat-tolerant genotypes with high *g*_S_ also had higher photosynthetic efficiency under severe heat stress; therefore, this trait acts as a useful genetic tool for developing heat tolerance. Stomatal conductance increased in heat-stressed tomato plants, relative to control conditions (Camejo et al., 2005). In another study, heat-tolerant tomato genotypes maintained higher stomatal conductance under stressed conditions (36/28°C), relative to the control (26/18°C). Further, heat stress severely affected stomatal anatomy and stomatal number in heat-sensitive genotypes, relative to heat-tolerant genotypes (Zhou et al., 2015).

Multiple screening parameters, including stomatal conductance, were used to screen 15 common bean genotypes for heat tolerance in a greenhouse chamber (Traub et al., 2018). Five genotypes—SB761, SB776, SB781, Jaguar, and TB1—were screened at three temperature regimes (35/30, 40/35, 45/40°C). Stomatal conductance increased with increasing temperature until 40/35°C—after which, it declined—genotype TB1 had the highest values for stomatal conductance. In mungbean genotypes, *g*_S_ increased up to 40/30°C but declined significantly under heat stress at 43/30°C and 45/32°C, contributing to a rise in leaf temperature (Kaur et al., 2015). In another study on mungbean, *g*_S_ was used to differentiate between heat-tolerant and heat-sensitive genotypes (Sharma et al., 2016). Using a similar approach, Sita et al. (2017a) identified heat-tolerant lentil genotypes (IG2507, IG3263, IG3745, IG4258, and FLIP2009) on the basis of stomatal conductance, with *g*_S_ increasing with increasing temperature up to 38/28°C in heat-tolerant genotypes. Heat-tolerant genotypes also had higher *g*_S_ values under late-sown than normal-sown conditions; in contrast, heat-sensitive genotypes were unable to maintain higher *g*_S_ under heat stress. In chickpea, heat-tolerant (ICC1356, ICC15614) and heat-sensitive genotypes (ICC4567, ICC5912) genotypes were selected on the basis of leaf and seed traits (Awasthi et al., 2014)—heat-tolerant genotypes maintained higher stomatal conductance and photosynthetic function than heat-sensitive genotypes under similar conditions and produced more seed yield. Evaluation of three varieties of tomato (Nagcarlang, Hybrid 61 and Moskvich) against combined heat and drought stresses (25–45°C; 20% irrigation; 2 days), revealed that genotype Hybrid 61 performed better by maintaining higher stomatal conductance and having lower leaf temperature than other two varieties (Nankishore and Farrell, 2016), suggesting this trait to be useful even under stress combinations.

### Carbon Isotope Discrimination (CID,Δ^13^C)

Carbon isotope discrimination has become an important tool for interpreting photosynthetic rate and water use efficiency (WUE) in plant species (Sheshshayee et al., 2003; Bindumadhava et al., 2011). ^12^C (98.89%) and ^13^C (1.11%) are the two stable carbon isotopes (non-radioactive) in the global carbon pool. Small but significant amount of ^13^C (heavy isotope) incorporated in the organic and inorganic matter during CO_2_ fixation by carboxylating enzymes. These small differences in ^13^C abundance are expressed as Carbon isotope ratio and analyzed with isotope ratio mass spectrometer (IRMS) (Farquhar et al., 1989). Composition of carbon isotopes in plant tissue samples show photosynthetic ability governed by RuBisCO in mesophyll tissues (Bindumadhava et al., 2005, 2011, Impa et al., 2005). Lower values of CID represent lower stomatal conductance (limited diffusion of CO_2_) and vice versa (Bindumadhava et al., 2011). Further, under high temperature, leaf water status declines due to reduced root hydraulic conductivity, resulting in stomatal closure (Hairat and Khurana, 2016). Therefore, lower CID values at high temperature can be ascribed to indicate declined root absorption and stomatal closure. In barley, carbon-13 discrimination is a useful indicator of high yield (Craufurd et al., 1999), and could be a sound screening parameter for identifying heat-tolerant genotypes. Heat-tolerant (C306, K7903) and heat-sensitive (HD2329) wheat genotypes were identified from CID values and other physiological traits. The heat-tolerant genotypes had higher mean CID values at high temperature (42°C) than the heat-sensitive genotypes. This study demonstrated that the heat-tolerant genotype maintained stomatal opening by accumulating osmolytes, such as proline, to maintain osmotic pressure for water absorption (Hairat and Khurana, 2016).

### Photosynthetic Pigments

Heat stress negatively affects photosynthesis by decreasing leaf pigment content and damaging leaf ultrastructure. Chloroplasts play a vital role in photosynthesis as one of the most heat-sensitive organelles (Krause and Santarius, 1975; Ogweno et al., 2008; Abdelmageed and Gruda, 2009). Decreases in total chlorophyll content and changes in the chlorophyll a/b ratio have been correlated with reductions in photosynthesis during heat stress, due to reduced “antenna (pigment units)” size and thus reduced light-harvesting (Blum, 1986; Harding et al., 1990; Shanmugam et al., 2013). The stay-green (SGR) trait, or delayed leaf senescence, is a crucial trait that allows plants to retain leaves in an active photosynthetic state under high temperature to maintain the assimilation process and increase crop yield (Gregersen et al., 2013; Kumari et al., 2013). Stay-green rice genotypes exhibited high photosynthetic activities under heat stress, resulting in high yields (Jagadish et al., 2015).

Chlorophyll content is an integrative trait that is correlated with stomatal conductance, photosynthetic rate, and transpiration (Del Blanco et al., 2000; Netto et al., 2005), and considered a good criterion for screening for heat-stress tolerance. In the current era of global climate change, introduction of the SGR trait is vital for developing heat-resistant cultivars (Kumari et al., 2013). The SGR trait has been linked to increased yield production in many crops under heat stress, including wheat, barley, rice, maize, and cowpea (Kumari et al., 2007; Borrell et al., 2014; Kobata et al., 2015; Gous et al., 2016; Abdelrahman et al., 2017). The stay-green trait has helped to identify heat-tolerant wheat cultivars that maintain yields at high temperatures (Vijayalakshmi et al., 2010). A significant correlation was detected between heat tolerance and the stay-green trait in 936 elite wheat genotypes (Kumari et al., 2007), suggesting that delayed senescence is an essential selection criterion for heat adaptability. The stay-green characteristic of wheat cultivar Mairaj-2008 was correlated with higher grain yield under heat stress than other lines that lacked the stay-green trait (Nawaz et al., 2013). Genotypes with delayed leaf senescence or stay-green traits have been associated with thermotolerance, due to the longer grain-filling period and thus higher yields, relative to genotypes lacking these traits (Reynolds et al., 1997; Vijayalakshmi et al., 2010). Delayed leaf senescence enhances the transpiration use efficiency, resulting in higher yields. Thus, the stay-green trait is beneficial for retaining active photosynthesis under heat stress (Bavei et al., 2011).

The stay-green trait was used to identify three promising heat-tolerant wheat genotypes [CB-367 (BB#2/PT//CC/INIA/3/ALD“S”), CB-333 (WL 711/3/KAL/BB//ALD “S”), and CB-335 (WL711/CROW “S”//ALD#1/CMH 77A] based on maximum grain development and survival under heat stress (32°C for 4 weeks) (Rehman et al., 2009). Two recombinant inbred lines (RILs) of wheat, SB062 and SB003, were exposed to 7-day heat shocks (32.7/21.6°C day/night) in a growth chamber during the vegetative or reproductive stage. SB062 maintained leaf greenness for longer than SB003 under heat stress and identified as heat-tolerant; in addition, delayed leaf senescence appeared to play a role in maintaining grain size in SB062 under heat stress (Ullah and Chenu, 2019). Lu et al. (1997) suggested that higher stomatal conductance and photosynthetic rate are functionally important for higher heat tolerance and yields. A high temperature (38/28°C) treatment for 6 days under controlled conditions in a greenhouse modified chlorophyll content in two contrasting maize genotypes; DTPYC9F119 maintained higher leaf chlorophyll content (identified as heat-stress tolerant) than K64R (identified as heat-stress susceptible) (Debnath et al., 2016; Singh et al., 2020). In another study, 12 barley genotypes were exposed to heat stress (> 40°C) for 107–119 days in the field—genotypes L3, L6, L8, and L10 had longer stay-green duration and higher yields under heat stress than the other genotypes. Fifteen cotton genotypes were screened for thermotolerance (40°C) in the field—genotypes AGC375 and AGC208 were identified as heat-tolerant based on their chlorophyll content (Karademir et al., 2012). In a similar study, cotton genotype Sicot 53 had higher thermotolerance than Sicala 45 (Cottee et al., 2007). In rice, cultivar N44 was identified as heat-tolerant (exposed to 38°C for 25 days in the field during the reproductive stage), with its higher chlorophyll content under heat stress than N-22 (Bahuguna et al., 2015).

Chlorophyll content was used to screen for heat tolerance in several lentil genotypes after exposure to heat stress (>32/20°C) in a growth chamber at the vegetative and reproductive stage. Heat-tolerant genotypes IG3263 and IG2507 had more chlorophyll than heat-sensitive genotypes IG4242 and IG3964, which was positively correlated with yield (Sita et al., 2017a). In chickpea, genotypes were selected for heat tolerance based on the SGR trait; plants were exposed to gradual increasing temperatures (2°C per day) from 27/18°C to 42/25°C day/night for 8 days in a growth chamber; at which time, genotype ICC16374 (heat-sensitive) had lower leaf chlorophyll content than JG14 (heat-tolerant) (Parankusam et al., 2017). Likewise, Kaushal et al. (2013) identified two heat-tolerant (ICC15614, ICCV92944) and two heat-sensitive (ICC10685, ICC5912) chickpea genotypes based on chlorophyll content, after exposure to heat stress (>32°C/20°C) in the field during reproductive development. The stay-green trait could be used as a morphological indicator for thermotolerance in tomato, as in wheat (Sharma D. K. et al., 2015; Zhou et al., 2015). The stay-green trait contributes to high yield in tomato exposed to heat stress (Zhou et al., 2015). Tomato’s ability to stay-green and maintain photosynthesis during heat stress at different developmental stages, especially anthesis, could be vital for reproductive growth and yield (Zhou et al., 2017). Heat-sensitive tomato genotypes do not stay-green under heat stress due to the decline in chlorophyll and carotenoid contents, and show early chlorosis and withered leaves (Vijayalakshmi et al., 2010; Zhou et al., 2015).

### Chlorophyll Fluorescence

Chlorophyll fluorescence (*F_*v*_/F_*m*_* ratio) is a relatively sensitive indicator of direct or indirect effects of abiotic stress on photosynthesis (Schreiber and Bilger, 1993). The relationships between primary photosynthetic reactions and chlorophyll fluorescence are crucial as they provide information on the plant’s photosynthetic capability and its acclimation capacity under stressful environmental conditions (Lichtenthaler, 1987; Kalaji et al., 2018). Of the photosynthetic apparatus, photosystem II (PSII) is the most heat-labile cell structure (Vacha et al., 2007). As damage to PSII is often the first response when plants are subjected to heat stress, PSII response studies can reveal the primary effects of heat stress on plants (Mathur et al., 2011; Van der Tol et al., 2014); measuring chlorophyll *a* fluorescence is an effective and non-invasive technique to identify damage to PSII efficiency (Baker and Rosenqvist, 2004; Baker, 2008). The ratio between variable fluorescence (*F*_v_) and maximum fluorescence (*F*_m_), or *F*_v_/*F*_m_, reflects the maximum quantum efficiency of PSII (Butler, 1978), and is one of the most heat-affected fluorescence parameters. A decline in *F*_v_/*F*_m_ is frequently observed when plants are subjected to abiotic stress, including heat (Willits and Peet, 2001; Molina-Bravo et al., 2011; Sharma et al., 2012). There is a negative linear correlation between *F_*v*_/F_*m*_* and the maximum quantum yield of photosynthesis, when measured as O_2_ evolution (Demmig and Björkman, 1987; Kao and Forseth, 1992) and CO_2_ fixation (Ogren and Sjostrom, 1990). Screening methodologies using chlorophyll fluorescence to detect and quantify damage in photosystem II (PSII) and thylakoid membranes in response to temperature stress have been used in several cereal crops, including barley (Rizza et al., 2011), wheat (Balouchi, 2010), maize (Sinsawat et al., 2004), legume crops [chickpea, groundnut, pigeon pea (*Cajnus cajan*), and soybean] (Srinivasan et al., 1996; Herzog and Chai-Arree, 2012), and horticultural crops, including strawberry (*Fragaria ananassa*) (Ledesma et al., 2004; Kadir et al., 2006), tomato (Willits and Peet, 2001), grapes (*Vitis vinifera*) (Kadir et al., 2007), and various tropical and subtropical fruits (Yamada et al., 1996; Weng and Lai, 2005). Therefore, chlorophyll fluorescence is a promising tool for detecting stress-induced injuries and thermotolerance (Méthy et al., 1994) but its successful implementation in crop breeding programs requires careful selection of suitable fluorescence parameters (Malaspina et al., 2014).

Heat-tolerant wheat lines with tolerance to high temperatures during grain filling had greater *F_*v*_/F_*m*_* ratios than heat-sensitive lines in warmer irrigated environments, which were linked to higher grain yield (Shefazadeh et al., 2012). The physiological state of thylakoid membranes, as determined by chlorophyll *a* fluorescence, identified heat-tolerant wheat cultivars with high chlorophyll fluorescence (Ristic et al., 2007). Various wheat lines were exposed to heat stress for 3 days at 40°C in controlled conditions; the lines having high chlorophyll fluorescence (*F_*v*_/F_*m*_* 0.836)—830, 1313, 1039, 1223—were less sensitive to heat in terms of growth and photosynthesis than the other lines, and were identified as heat-tolerant (Sharma et al., 2014). Similarly, genotypic variation for chlorophyll fluorescence parameters exists in rice under heat stress (29°C for 25 days at anthesis) in a growth chamber; N22 genotype maintained high *F_*v*_/F_*m*_* (0.75) under heat stress, and was identified as heat-tolerant, relative to the low *F_*v*_/F_*m*_* (0.70) in Vandana (Sailaja et al., 2015). Modified chlorophyll fluorescence imaging was used to screen 20 wild barley (*Hordeum spontaneum*) genotypes exposed to heat stress (45°C, 1 h) in growth chambers, and identified HOR10478 as the most heat-sensitive and HOR12818 as the most heat-tolerant genotypes (Jedmowski and Brüggemann, 2015). Oukarroum et al. (2016) also differentiated heat tolerance in 10 varieties of barley. After 2 weeks of growth, detached leaves were exposed to a short-term heat treatment at 45°C for 10 min in a growth chamber, which decreased chlorophyll fluorescence; notably, varieties Ig, Im, and Tz had high chlorophyll fluorescence (heat-tolerant) and Ma, Ra and I_gr_ had low chlorophyll fluorescence (heat-sensitive).

In many legumes, chlorophyll fluorescence has been used to identify genotypes that tolerate heat stress. In lentil, photosynthetic efficiency was measured as PSII function (*F_*v*_/F_*m*_* ratio) in a natural environment by exposing plants to heat stress (above 32/20°C) during the reproductive stage. Heat-tolerant genotypes—IG2507, IG3263, IG3297, IG3312, IG3327, IG3546, IG3330, IG3745, IG4258, and FLIP2009—maintained high chlorophyll fluorescence (*F_*v*_/F_*m*_* 0.71) under heat stress, relative to heat-sensitive genotypes—IG2821, IG2849, IG4242, IG3973, IG3964—which had the lowest *F_*v*_/F_*m*_* values (0.58) (Sita et al., 2017a). Nine common bean lines were measured for changes in chlorophyll fluorescence under heat stress at flowering (2 h at 45°C) in a greenhouse; thermotolerant lines 83201007 and RRR46 had higher *F_*v*_/F_*m*_* values under heat stress than the heat-sensitive line Secuntsa (Petkova et al., 2009). In another study, 12 varieties and lines of common bean were exposed to 42°C in the field during the reproductive period; two genotypes (Ranit and Nerine) maintained *F_*v*_/F_*m*_* values at 42°C, relative to the controls at 26°C, and were considered heat-tolerant. These two genotypes also showed good productivity and quality and can be used as parental lines in bean breeding programs (Petkova et al., 2007). Likewise, 41 mungbean lines were grown outdoors and exposed to high temperatures (>40/28°C) during the reproductive stage; several promising heat-tolerant lines (EC693358, EC693357, EC693369, Harsha, and ML1299) were identified, with high *F_*v*_/F_*m*_* ratios (0.73–0.75 units) compared to sensitive lines (0.61–0.67 units), which would not only serve as useful donor/s for breeding programs, but also as suitable base plant source to gain insight into heat-stress-induced effects in cell metabolism (Sharma et al., 2016). In chickpea, heat stress (>30°C) in the field during the reproductive stage reduced *F_*v*_/F_*m*_* more (0.48, 0.41) in two heat-sensitive genotypes ICC10685 and ICC5912, than in two heat-tolerant genotypes ICC15614 and ICCV92944 (0.64, 0.60) (Kaushal et al., 2013; Awasthi et al., 2014). A field experiment conducted in two winter seasons at three locations with known differences in temperature in NE South Africa, involving four chickpea genotypes, showed. that two genotypes, which were tolerant to heat stress had chlorophyll fluorescence (Fv/Fm) of 0.83–0.85 at the warmer site, while the two sensitive genotypes showed lower Fv/Fm of 0.78–0.80; these values correlated positively with grain yield. The two tolerant genotypes had higher photosynthetic rates, starch, sucrose and grain yield than the sensitive genotypes at the warmer site. The observation revealed that chlorophyll fluorescence and leaf carbohydrates are suitable tools for selection of heat tolerant chickpea genotypes under field conditions (Makonya et al., 2019). Screening of 15 alfalfa (*Medicago sativa* L.) genotypes by exposing seedlings to 38/35°C day/night for 7 days in a growth chamber identified Bara310SC (*F_*v*_/F_*m*_* 0.79) and WL712 (*F_*v*_/F_*m*_* < 0.79) as heat-tolerant and heat-sensitive cultivars, respectively (Wassie et al., 2019), showing that *F_*v*_/F_*m*_* is an effective tool for phenotyping contrasting genotypes for heat tolerance.

The heat susceptibilities of 67 tomato genotypes were evaluated in a climate chamber—the genotypes with higher *F*_*v*_/*F*_m_ under heat stress (36/28°C for 4 days or 40°C for 7 h), maintained their physiological status, relative to genotypes with lower *F*_v_/*F*_m_ (Zhou et al., 2015). The two genotypes with the highest *F_*v*_/F_*m*_* ratios (heat-tolerant group; T1, T2; 0.82, 0.80 units) and two with the lowest *F_*v*_/F_*m*_* ratios (heat-sensitive group; S1 and S2; 0.74, 0.77 units) were selected for further study (Zhou et al., 2015). Another study screened wild genotypes and cultivars of tomato in a growth chamber at 33°C—wild tomato varieties Pe and Pr1 had the highest temperature stress tolerance with high *F*_v_/*F*_m_ ratios (0.56, 0.58), while the cultivated species were more sensitive to temperature stress with lower *F*_v_/*F*_m_ ratios (0. 28, 0.38) (Zhou et al., 2018).

Chlorophyll fluorescence was used to screen cotton landraces—6-week-old cotton plants were subjected to heat stress at 45°C in a growth chamber to determine thermotolerance in terms of photosynthetic ability, independent of agronomic yield and productivity. Three genotypes (TX2287, TX2285, and TX761) maintained high photosynthetic efficiency (*F_*v*_/F_*m*_* 0.57), relative to sensitive genotype (*F_*v*_/F_*m*_* 0.46) (Wu et al., 2014). In another growth chamber study, a commercial set of eight cotton genotypes was screened for heat tolerance by subjecting to heat stress (>35°C); four genotypes (SG215BR, ST474, and DP444BG/RR) had relatively high *F_*v*_/F_*m*_* indicating that they suffered less from stress, while Sphinx and Acala Riata had low *F_*v*_/F_*m*_*, indicating temperature sensitivity (Bibi et al., 2004). In a related study, screening of 15 cotton genotypes for thermotolerance (40°C) in the field identified genotypes AGC375 and AGC208 as heat-tolerant, based on their superior chlorophyll fluorescence (Karademir et al., 2012). Imposing combined drought and heat stress significantly affected the photosynthetic efficiency of chickpea (*Cicer arietinum*) genotypes, in a study conducted in outdoor conditions at two different sowing times [November (<32–20°C at the time of reproductive stage; control) and in February (>32–20°C at the time of reproductive stage; heat stress during pod filling)], while drought was applied during both sowing times during pod filling (at ∼75% podding) by withholding water until maturity. The photosynthetic efficiency (Fv/Fm) of the leaves decreased more in plants subjected to drought stress (54–74%) than to heat stress alone (9–46%) and the combined heat + drought stress treatment showed the greatest reduction in photosynthetic efficiency (68–83%), with the smallest reduction occurring in the drought-tolerant genotype (ICC8950), compared to drought-and heat sensitive genotypes (Awasthi et al., 2017).

### Photosynthetic Rate

Heat stress affects plant characteristics such as the stay-green trait, chlorophyll content, and chlorophyll fluorescence, which influences the photosynthetic rate (Sharkey, 2005). Hence, photosynthetic rate can be used as a screening parameter for the selection of heat-tolerant genotypes. Variation in photosynthetic rate among plant species in response to heat stress has been well-documented. For example, a heat-shock treatment (45°C for 2 h at the fourth true leaf stage) reduced the net photosynthetic rate (*P*_n_) of two tomato cultivars, more so in Campbell-28 (heat-sensitive) than wild Nagcarlang (heat-tolerant) (Camejo et al., 2005). High temperature deactivates RuBisCo, which could be involved in reducing photosynthetic rate (Sharkey, 2005). Another study on tomato compared the *P*_*n*_ of one cultivated (Ly from *Solanum lycopersicum*) and six wild (Ha from *S. habrochaites*, Pe from *S. pennellii*, Pi1 and Pi2 from *S. pimpinellifolium*, Pr1 and Pr2 from *S. peruvianum*) genotypes grown at high temperature (33°C) in a growth chamber—Ly, Ha, Pi1, and Pi2 had lower *P*_*n*_ than the control, while Pe, Pr1, and Pr2 showed higher *P*_*n*_ indicating their heat tolerance (Zhou et al., 2018). Plants of the tomato cultivar “Liaoyuanduoli” grown in greenhouse exposed to heat stress (35°C after 15 DAS led to a significant change in photosynthetic apparatus as damage of chloroplast membrane and at the same time, the thylakoids loosely distributed with lesser grana, thus, changed chloroplast ultrastructure might have declined the *P*_*n*_ (Zhang et al., 2014). In rice, heat tolerant genotype (N22) could maintain photosynthetic activity for a longer time after anthesis and thus could produce higher grain weights, compared to heat-sensitive genotypes (IR20, IR53, IR46) (Gesch et al., 2003).

Soybean cultivars (IA3023 and KS4694) and PI lines (PI393540 and PI588026A) expressed heat tolerance and susceptibility with high and low *P*_n_, respectively (Djanaguiraman et al., 2019). The soybean cultivars had less thylakoid membrane damage than the PI lines. In an earlier study on soybean genotype K03−2897, high-temperature stress (38/28°C) for 14 days at the flowering stage significantly decreased leaf *P*_n_, due to anatomical and structural changes (increased thickness of palisade and spongy layers and lower epidermis) in cells and cell organelles, particularly damage to chloroplasts and mitochondria (Djanaguiraman and Prasad, 2010). Two heat-tolerant chickpea genotypes (Acc#RR-3, Acc#7) had higher *P*_n_ than two heat-sensitive genotypes (Acc#2, Acc#8) at high temperature (35/30°C), which may have been due to increased RuBisCo activity (Makonya et al., 2019). In another chickpea study, 56 genotypes were exposed to high temperatures in the field from the flowering stage to crop maturity (maximum temperatures 25–40°C)—the tolerant genotypes (PUSA1103, PUSA1003, KWR108, BGM408, BG240, PG95333, JG14, BG) had higher Pn than the sensitive genotypes (ICC1882, PUSA372, PUSA2024) (Kumar et al., 2017). Similarly, the response of four chickpea genotypes to a natural temperature gradient in the field at the flowering stage identified two heat-tolerant genotypes (Acc#RR-3, Acc#7) with high *P*_n_ and two heat-sensitive genotypes (Acc#2, Acc#8) with lower *P*_n_; these results were validated in a climate chamber experiment set at 30/25°C and 35/30°C (Makonya et al., 2019). Improvement of heat stress tolerance by stabilizing PSII system through introducing *IbOr* gene in transgenic potato (Goo et al., 2015), sweet potato (Kang et al., 2017), and in alfalfa (Wang et al., 2015) is worth mentioning. Heat, drought and their combination limited the Photosynthetic rate of lentil (*Lens culinaris* Medikus), particularly during reproductive growth and seed filling. In recent study eight lentil genotypes two drought-tolerant (DT; DPL53 and JL1), two drought-sensitive (DS; ILL 2150 and ILL 4345), two heat-tolerant (HT; 1G 2507 and 1G 4258) and two heat-sensitive (HS; 1G 3973 and 1G 3964) sown at the normal time (November), at the time of seed filling (30/20°C), or sown late (February) to impose heat stress (> 30/20°C (day/night) and drought maintained by water withheld (50% of field capacity) from the start of seed filling to maturity. The photosynthetic rate (Pn) decreased significantly more under drought stress (33.4–56.6%) than heat stress (13.3–43%), as compared to the control plants. Under the combined stress, Pn declined more (57–82% reduction), less so in the heat and drought tolerant genotypes compared to sensitive (Sehgal et al., 2017).

### Sucrose

Leaf photosynthates are largely transported to sink organs in the form of sucrose, and sucrose synthase (SS) is a key enzyme for sucrose to enter a variety of metabolic pathways (Lu et al., 2005). Down-regulation of SS indirectly inhibits carbohydrate production, eventually reducing yield and quality. Maintaining sucrose levels is vital during stressed conditions, which depend on its synthesis and hydrolysis. Heat-stressed plants had significant reductions in the activity of key enzymes—sucrose phosphate synthase (SPS) and SS—involved in sucrose synthesis. The availability of sucrose to reproductive organs is crucial for sustaining their function (Kaushal et al., 2013). Heat-tolerant genotypes are expected to stabilize the photosynthetic process better than heat-sensitive genotypes. Measuring sucrose concentrations reveals the photosynthetic status of plants under heat stress (Awasthi et al., 2014). A large core-collection of chickpea genotypes screened or heat tolerance (32/20°C) in a natural environment identified two heat-tolerant (ICC15614, ICCV92944) and two heat-sensitive (ICC10685, ICC5912) genotypes. The heat-sensitive genotypes had significantly greater inhibition of RuBisCo (carbon-fixing enzyme), SPS, and SS than the heat-tolerant genotypes, and thus produced less sucrose than the tolerant genotypes (Kaushal et al., 2013). Heat-sensitive genotypes produced far less leaf sucrose than heat-tolerant genotypes, which impaired its supply to developing reproductive organs (flowers, pods, and seeds) in sorghum (Prasad and Djanaguiraman, 2011), tomato (Li et al., 2012), and chickpea (Kaushal et al., 2013).

In wheat, heat-tolerant genotypes (PBW343 and C306) exposed to heat stress (>25°C) in the field had higher SS activity and thus higher sucrose contents in grain than heat-sensitive genotypes (PBW521, PBW522) (Bavita et al., 2012). Limitations in sucrose supply may disrupt the development and function of reproductive organs (Prasad and Djanaguiraman, 2011; Snider et al., 2011). In lentil, sucrose production is vital for leaf and anther function, and has been correlated with SPS activity in natural high-temperature environments (> 32/20°C). Heat-tolerant lentil genotypes (IG2507, IG3263, IG3297, IG3312, IG3327, IG3546, IG3330, IG3745, IG4258, and FLIP2009) produced more sucrose in their leaves (65–73%) and anthers (35–78%), than heat-sensitive genotypes (IG2821, IG2849, IG4242, IG3973, IG3964), which was associated with superior reproductive function and nodulation in tolerant genotypes (Sita et al., 2017a). Thus, heat stress negatively affects sucrose metabolism due to the inhibition of carbon fixation and assimilation (Awasthi et al., 2014). Sucrose concentrations in leaves and anthers and SS and SPS activities declined significantly in two mungbean genotypes (SML832 and SML668) exposed to heat stress (>40/25°C day/night) outdoors and in a controlled environment, more so in SML668 (heat-tolerant) than SML832 (heat-susceptible) (Kaur et al., 2015). Tomato cultivars exposed to heat stress in growth chambers (31/25°C day/night) or greenhouses (32/26°C day/night) revealed four genotypes (FLA7516, Hazera3018, Hazera3042, and Saladate) as heat-tolerant with high sucrose contents in the mature pollen grains, and three genotypes (Grace, NC8288, and Hazera3017) as heat-sensitive, with 50% less sucrose than the tolerant genotypes (Firon et al., 2006).

Expression of the sucrose transporter gene, OsSUT1, is important for maintaining photo-assimilate supply to grains. In rice exposed to high-temperature stress (31/26°C) in a glasshouse, cultivar Genkitsukushi (heat-tolerant) had higher expression of OsSUT1 in stems than Tsukushiroman (heat-sensitive), indicating that sugar transport is more effective in Genkitsukushi than Tsukushiroman under heat stress, which improves grain quality (Miyazaki et al., 2013).

## Biochemical Traits

Heat sensitivity is linked to the expression of several cellular molecules, including antioxidants (Wilson et al., 2014), HSPs (Xu et al., 2011) osmolytes (Bita and Gerats, 2013), and phytohormones (Sharma et al., 2020). These molecules assist cells to adapt, repair, and survive in adverse temperature environments; hence, measuring the extent of their expression in contrasting genotypes grown under heat stress might reveal mechanisms regulating the heat response.

### Oxidative Stress and Antioxidants

Heat stress negatively affects cellular metabolism due to extensive ROS production that can severely damage lipids, proteins, and nucleic acids (Bita and Gerats, 2013). Plants protect themselves from ROS production by activating enzymatic and non-enzymatic processes (Bita and Gerats, 2013). The main ROS-scavenging enzymes are superoxide dismutase (SOD), catalase (CAT), peroxidase (POD), ascorbate peroxidase (APX), and glutathione reductase (GR), and the non-enzymatic system includes ascorbic acid (ASC) and glutathione (GSH) (Suzuki et al., 2012). Genotypes can be selected based on their enzyme expression level, with more prominent activities among heat-tolerant than heat-sensitive genotypes (Kumar et al., 2013). Genotypes respond differently to heat stress due to variation in their antioxidant systems. Hence, this trait is useful for identifying heat-tolerant genotypes.

Two tomato cultivars differing in heat sensitivity (Sufen14, Jinlingmeiyu) were raised in a greenhouse in optimum temperature (26/18°C) and heat-stressed (38/30°C for 6 days with 2 days recovery). Jinlingmeiyu had lower activities of SOD, POD, APX, and MDA (malondialdehyde) and lower proline content than Sufen14, suggesting the involvement of these enzymes in imparting heat tolerance in Sufen14 (Zhou et al., 2019). Categorization of 50 *Brassica juncea* genotypes into tolerant, moderately tolerant and susceptible genotypes after exposure to 45°C was based on oxidative damage tolerant genotypes had less lipid peroxidation and higher POD, CAT, and GR activities than moderately tolerant and susceptible genotypes (Wilson et al., 2014). In contrast, *Brassica juncea* seedlings grown under optimum (25°C) and high (45°C) temperatures had higher MDA and lipoxygenase (LOX) activities of antioxidants (SOD, CAT, POX, APX, and GR) in the thermosensitive genotype (NPJ-119) than the thermotolerant genotype (NRCDR-02) suggesting variations in the response of antioxidatnts, which might be stage-or plant-specific (Rani et al., 2012). Wheat genotypes were differentiated into heat-tolerant (C306), intermediate heat-tolerant (HD2285), and heat-sensitive genotype (HD2329) by subjecting them to heat stress (8 and 23 days after anthesis) by delaying the sowing time: C306 had higher relative water content, ASC, APO, CAT, and SOD and lower lipid peroxidation and H_2_O_2_ content than HD2285 and HD2329 (Sairam and Srivastava, 2000).

In chickpea plants raised under natural conditions and heat stressed at 50% flowering (30/20, 35/25, 40/30, and 45/35°C) in growth chambers, tolerant genotypes (ICCV07110, ICCV92944) had lower MDA concentration and H_2_O_2_ content than sensitive genotypes (ICC14183, ICC5912), which was attributed to their higher activity levels of APX, GR, and ASC (Kumar et al., 2013). Forty-one mungbean genotypes exposed to heat stress (>40/28°C) in the field revealed that heat-tolerant genotypes (EC693357, EC693358, EC693369, Harsha, and ML1299) suffered less oxidative damage (1.52–2.0-fold increase MDA; 1.59–1.96-fold increase H_2_O_2_) than sensitive genotypes (2.2–2.4-fold increase MDA; 2.21–2.93-fold H_2_O_2_) (Sharma et al., 2016). The heat-tolerant genotypes also significantly increased APX activity (by 1.48–1.77-fold), relative to susceptible genotypes (1.27–1.37-fold) and similar response was observed for GR activity. However, heat-tolerant and heat-sensitive genotypes had similar increases in CAT activity. Similarly, 38 lentil accessions screened for heat tolerance (>35/20°C) during the reproductive stage revealed less oxidative damage (MDA and H_2_O_2_ contents increased) and higher SOD, CAT, APX, and GR activities—involved in detoxification—in heat-tolerant genotypes (IG2507, IG3263, IG3745, IG4258 and FLIP2009) than heat-sensitive genotypes (IG2821, IG2849, IG4242, IG3973, IG3964 (Sita et al., 2017a). Concurrence of heat and drought stress will do more damage at the biochemical level. Oxidative damage and antioxidant mechanisms responding toward combined stress were reported in tomato cultivars. Two cultivars of tomato (CV1; Sufen14 and CV2; Jinlingmeiyu) were raised in green house conditions to compare the cultivar difference. Treatment (Heat stress-38/30°C, and drought stress-no irrigation) were given to 28 days old seedlings for six days. Significant increase in ROS such as H_2_O_2_ and O^2–^ were reported in both the cultivars than control (26/18°C). Their studies showed that CV2 had lower activity of enzymes-peroxidase, ascrobate peroxidase, superoxide dismutase, malondialdehyde (MDA) and proline content than CV1, under combined stress on day 6, clearly depicting cultivar differences with respect to antioxidant activity (Zhou et al., 2019).

### Metabolites

Plant metabolites are low molecular weight compounds involved in stress tolerance. They play a crucial role in maintaining the redox homeostasis of cells and stabilizing cell membranes and proteins (Wahid et al., 2007) through various intermediate/precursor compounds, such as compatible solutes, signaling agents, and antioxidants (Kaplan et al., 2004). Metabolites are categorized into primary and secondary metabolites. Primary metabolites that are specifically upregulated in response to abiotic stress are amino acids (proline), polyamines (spermidine, spermine, putrescine), carbohydrates (sucrose, hexoses, polyhydric alcohols), and glycine betaine. Similarly, secondary metabolites include phenolic compounds (flavonoids, isoflavonoids, anthocyanins), terpenoids (saponins, tocopherols), and nitrogen-containing metabolites (alkaloids and glucosinolates) (Rodziewicz et al., 2014). Under heat stress, plants restructure their metabolites to help the cells to maintain homeostasis via the production of stress-induced compounds (Serrano et al., 2019). Activation of heat-shock factors, such as HSFA2 and HSFA3, increases the level of metabolites such as galactinol and its derivatives in response to heat stress (Song et al., 2016). Therefore, metabolites may serve as a useful tool for selecting heat-tolerant varieties under high-temperature stress. Comparing heat-tolerant and heat-sensitive genotypes can identify metabolite markers that are constitutively expressed and allow selection of superior germplasm.

Seed metabolomic analysis performed on contrasting soybean genotypes (PI587982A, heat-tolerant; A5279 and DP3478, heat-sensitive) revealed 25 metabolites that differed between genotypes, including tocopherol isoforms, ascorbate precursors, flavonoids, two amino acids, and amino acid derivatives (Chebrolu et al., 2016). At 36°C, 10 flavonoids were more abundant in the seeds of the heat-tolerant genotype than the heat-sensitive genotypes, along with several tocopherols (major antioxidants). Moreover, the heat-tolerant genotype had higher levels of a precursor of L-ascorbic acid biosynthesis—gulono-1,4-lactone—than the heat-tolerant genotypes. Overexpression of these stress-induced compounds provides thermotolerance to soybean seeds, which ultimately perform better in terms of seed vigor, seed germination, seed weight, and oil content. Metabolomic analysis of rice spikelets in a heat-tolerant (N22) and heat-sensitive (Moroberekan) genotype revealed that N22 accumulated more metabolites than Moroberekan, including carbohydrates (glucose 6-phosphate, fructose 6-phosphate, glucose, maltose, and other sugars), compatible solutes, and amino acids (leucine, isoleucine, and valine). N22 had lower levels of trehalose, sugar phosphatases, malic acid, and galactaric acid than Moroberekan under heat stress (Li X. et al., 2015). In wheat, a comparative analysis of metabolites in transgenic wheat (PC_27_ and PC_5_) and its wild type (varying in heat sensitivity), exposed to heat stress (40°C for 4 h) during heading revealed 25 metabolites that were highly expressed in transgenic wheat, including proline, three sugar alcohols (inositol, mannitol, and xylitol), pyruvic acid, and other amino acids (glycine, alanine, serine, valine, and tyrosine) (Qi et al., 2017). The metabolite profiling approach is an effective way to accurately screen and select the best-performing genotypes.

Proline is a multifunctional amino acid with diverse roles in maintaining cellular redox balance by dissipating excess of reducing potential (Rivero et al., 2004). Proline levels are upregulated under stress conditions as its biosynthesis is an adaptive response to reduce excess NADPH produced in response to the halt in CO_2_ fixation in the Calvin cycle due to stomatal closure (Berry and Bjorkman, 1980). Moreover, under stress conditions, proline is involved in osmotic adjustment, ROS scavenging, and as an energy source. Therefore, high proline contents under high-temperature stress can be used to screen heat-tolerant genotypes. Twenty wheat genotypes were screened for heat tolerance by exposing them to 25 or 35°C, and measuring proline content and membrane damage (Ahmed and Hasan, 2011). Heat-tolerant genotypes (Bijoy, Sufi, Kanchan, Fang 60, BAW 1059, BL 1883, BL 1022, IVT 7, IVT 8, IVT 9, IVT 10, and BAW 917) had higher proline contents (>200%) and less membrane damage (<50%) than heat-sensitive genotypes (Shatabdi, PRODIP, BAW 1064, Gourab, Pavon 76, Sonara, Kalyansona, and IVT 6). Thirty-five-day-old seedlings of different cabbage cultivars, including Chinese cabbage and their hybrids, were exposed to two temperature regimes (25 or 35°C) at the flower bud stage and assessed for heat tolerance based on proline contents in stalks, flower buds, and leaves—heat-tolerant cultivars (Yoshin, Kenshin, and full white) had higher proline levels than heat-sensitive cultivars (YR Kinshun, Chihiri 70, and Large leaf) (Hossain et al., 1995). Six cotton cultivars (Sicala, Acala 1517-88, Molopo, Alpha, Delta Pine Acala90, and OR19) were tested for genetic variability against combined heat and drought stress. Stress treatment (Heat stress; 40°C without irrigation for 15 days) were imposed on 3 weeks old seedlings. Stress treatment were increased the proline content in all the genotypes but the accumulation was more in tolerant genotypes (Alpha, Delta Pine Acala90, and OR19) compared to sensitive genotypes (Sicala, Acala 1517-88, Molopo) (De Ronde et al., 2000).

### Heat-Shock Proteins

During rapid heat stress, plants synthesize and accumulate specific proteins called heat-shock proteins (HSPs) (Howarth, 1991); this is a universal response to high-temperature stress in all organisms (Vierling, 1991). Heat-shock genes are upregulated during stress to encode HSPs which are vital for plant survival under such conditions (Chang et al., 2007). Three classes of HSPs are distinguished, according to molecular weight—HSP90, HSP70, and low molecular weight proteins. HSPs provide stress-related chaperone functions in plants under stress conditions (Reddy et al., 2010, 2016). Chaperones have a role in protein synthesis, maturation, targeting, degradation, renaturation, and membrane stabilization (Reddy et al., 2014, 2016). HSPs are located in the cytoplasm, nucleus, mitochondria, chloroplasts, and endoplasmic reticulum (Waters et al., 1996). Heat-stress transcription factors (HSFs), located in the cytoplasm in an inactive state, control HSP gene transcription and play a vital role in plant thermotolerance. Specific HSPs have been identified in response to high temperature, including HSP68 in the mitochondria of potato, maize, soybean, and barley (Neumann et al., 1994). The expression profiles of HSPs have been compared in plant species/genotypes contrasting in heat sensitivity. For instance, the higher heat tolerance of maize than wheat and rye at 42°C is correlated with the expression of five mitochondrial low molecular weight HSPs (28, 23, 22, 20, and 19 kDa), as opposed to only 20 kDa in wheat and rye (Korotaeva et al., 2001). According to Sharma-Natu et al. (2010), HSP18 was upregulated in developing grains of heat-tolerant wheat exposed to 3.2–3.6°C higher temperatures than normal. In other studies, HSP100 increased with heat stress in a tolerant wheat variety (Sumesh et al., 2008). Similarly, HSP26 increased in heat-tolerant wheat genotypes (K7903, C306) at 42°C, relative to heat-sensitive genotypes (PBW343, HD2329) (Hairat and Khurana, 2016). At 42°C, the expression levels of five Hsps—Hsp26.7, Hsp23.2, Hsp17.9A, Hsp17.4, and Hsp16.9A—were upregulated in the heat-tolerant rice cultivar Co39, relative to the heat-sensitive rice cultivar Azucena, and regarded as biomarkers for screening rice cultivars for heat tolerance (Chen et al., 2014). At 40°C, potato cultivar Norchip synthesized small (sm) Hsps for longer than other cultivars. In Norchip and Desiree, an 18 kDa small (sm)HSP increased for up to 24 h, while in cultivars Russet Burbank and Atlantic, the levels started to decline after 4 and 12 h respectively (Ahn et al., 2004). Anthers of a heat-tolerant tomato cultivar had higher constitutive levels of HSP100 than a heat-sensitive cultivar (Pressman et al., 2007). In chickpea, HSP levels increased in genotype JG14 (heat-tolerant) more than genotype ICC16374 (Heat-sensitive) when exposed to 42/25°C at anthesis (Parankusam et al., 2017). Likewise, in peanut, the best-characterized aspect of acquired thermotolerance is HSP production, with ICGS76, COC038, and COC068 selected as heat-tolerant genotypes and COC812, COC166, Tamrun OL 02, and Spanco selected as heat-sensitive (Selvaraj et al., 2011). In another study, heat-tolerant peanut genotype ICGS 44 showed higher HSP expression throughout the stress period than heat-sensitive genotypes AK 159 and DRG 1 (Chakraborty et al., 2018). Comparison of expression of heat shock proteins in wheat cultivars (Katya and Sadovo) under combined heat and drought stress (40°C/56%) reported 100% elevation of HSP100 and HSP70 as compared to 60 and 10% elevation under individual drought and heat stress in tolerant cultivar Katya (Grigorova et al., 2011).

## Gene/s Expression

Relatively few studies have been undertaken on changes in gene expression in contrasting genotypes under heat stress, but vital information has been garnered. In wheat, two contrasting genotypes—Chinese spring (heat-sensitive) and TAM107 (heat-tolerant)—were analyzed for changes in gene expression upon exposure to heat stress (40°C), using Affymetrix Barley 1 GeneChip, and expressed sequence tags. The analysis identified 6550 heat-responsive probe sets, accounting for 11% of the total probe sets (Qin et al., 2008). Heat-tolerant genotype (2199 probe set) had more heat-responsive probe sets than the heat-sensitive genotype (2084 probe set), which mainly belonged to HSPs, transcription factors, calcium and sugar signaling pathways, phytohormones biosynthesis and signaling, ribosomal proteins, RNA metabolism, and primary and secondary metabolites (Qin et al., 2008). In rice, contrasting genotypes N2219379 (heat-tolerant) and IR64 and N226264 (heat-susceptible) were compared for their heat response at 38°C using reproductive function and molecular approaches (González-Schain et al., 2016). Heat stress impaired reproductive functions, such as pollen production, pollen number, anther dehiscence, pollen germination, and stigma receptivity, more so in the sensitive genotypes than the tolerant genotype (Devasirvatham et al., 2012). Eighteen heat-responsive genes, such as HSFA2a, OsFKBP62b, and OsHSP17.9A had higher upregulation in tolerant genotypes than sensitive genotype. Under heat stress, the expression of HSFA2a increased 268-, 15-, and 3.2-fold in N2219379 (heat-tolerant), N226264 (heat-sensitive), and IR64 (heat-sensitive), respectively and that of OsFKBP62b increased by 108-, 10-, and 3-fold in N2219379, N226264, and IR64, respectively (González-Schain et al., 2016). A study was conducted on 197 spring wheat genotypes from ICARDA at two different locations, one in Sudan (Wad Medani) and another one in Egypt (Sids), to identify single nucleotide polymorphism (SNP) markers association mapping. The study detected 111 significant marker-trait associations; the wsnp_Ex_c12812_20324622 marker on chromosome 4A was significantly correlated with yield at both locations. Wheat genotypes carrying the cytosine base at the wsnp_Ex_c12812_20324622 and wsnp_Ex_c2526_4715978 markers produced more yield, compared to those carrying the alternative bases, by 15%, indicating the significance of involving these markers for marker-assisted selection in breeding programs to increase yield under heat stress. The best performing 20 high-yielding as well as heat-tolerant wheat genotypes, found in this study, have been distributed across Central and West Asia and North Africa (CWANA) and sub-Saharan Africa (SSA) for potential direct release and/or use as parents after local adaptation trials (Tadesse et al., 2019).

DNA methylation is one of the mechanisms of epigenetic modifications that plays a crucial role in imparting stress tolerance for various environmental stresses (Lukens and Zhan, 2007). A study on heat-tolerant (Huyou 2) and heat-sensitive (Fengyou 1) *Brassica napus* seedlings exposed to 45°C measured changes in DNA methylation levels and the cytosine methylation pattern using Methylation Sensitive Amplification Polymorphism (MSAP) analysis and RT-PCR (Gao et al., 2014). Under heat stress, percentage of methylated bands was 10.7% in Fengyou 1 (heat-sensitive) and 0.6% in Huyou 2 (heat-tolerant) (Gao et al., 2014). The cytosine methylation was also higher in the heat-sensitive genotype than the heat-tolerant genotype suggesting involvement of methylation to heat stress sensitivity. It has already been reported that superior crop genotypes avoid the methylation process (Gao et al., 2014). The effects of combined heat and drought stress on the gene expression in durum wheat (*Triticum turgidum subsp. durum*) cultivar “ofanto” were evaluated (Rampino et al., 2012). Plants were raised in the growth chamber and stress conditions were introduced at booting stage; heat stress- 30/22°C for 2 days, then raised to 34/24°C for following 2 days, 40/32°C for next one day and 42°C for last day and collected samples after 6 h of heat treatment, however, drought conditions were maintained at 28% field capacity. Gene expressions, analyzed through cDNA-AFLP studies, showed that combined stress down-regulated 92 genes and up-regulated 132 genes. Many of these genes reported to control the expression level of HSPs and dehydrins.

## Pollen-Based Traits

In most plant species, reproductive tissues, mainly male gametophytes, are more sensitive to heat stress than female gametophytes (Djanaguiraman et al., 2018a), and the threshold temperature for imposing damage in these tissues is lower than that for vegetative tissues. Damage imposed by heat stress can occur pre- or post-pollination, which impair fertilization and ultimately reduce seed set (Prasad et al., 2008a, 2017; Prasad and Djanaguiraman, 2014; Sage et al., 2015). Pre-pollination events that are highly susceptible to high temperature are (1) meiosis I and meiosis II of the microspore mother cell (Young et al., 2004), (2) development and subsequent dissolution of the tapetum layer (Farooq et al., 2011), and (3) exine and intine formation (Nahar et al., 2016). Post-pollination events that are highly susceptible to heat stress are (1) pollen load (Prasad et al., 1999b, 2006), (2) pollen germination (Prasad et al., 2001), (3) pollen tube growth (Prasad et al., 2001), and (4) fertilization (Prasad et al., 2001; Barnabás et al., 2008; Hedhly, 2011; Sita et al., 2017b). The development of male gametophyte under high temperature is more susceptible than female gametophyte (Djanaguiraman et al., 2018a; Liu et al., 2019). However, in peal millet (*Pennisetum glaucum*), the female gametophyte was more sensitive than male gameophyte (Djanaguiraman et al., 2018b). Several effects of heat stress on reproductive function have been reported. For instance, it reduced the fertility of the microgametophyte in *Brassica* (Rao et al., 1992), and impaired meiosis in the male gametophyte in tomato (*Lycopersicon esculentum*), which affected pollen germination and pollen tube growth (Firon et al., 2006). Shriveled pollen grains under high temperature may be why heat stress prevents starch accumulation in anther walls and pollen grains by disturbing the source–sink relationship that subsequently leads to lower levels of soluble sugars for their development (Pressman et al., 2002; Djanaguiraman et al., 2018a). Variation in contrasting genotypes of various pollen traits; could be used to identify and screen genotypes tolerant to high-temperature stress. For instance, 12 field-grown cultivars of *Brassica napus* L. were screened for heat tolerance based on pollen traits—pollen viability, pollen germination and pollen tube length—at 33.7°C (Singh et al., 2008). Pollen grains were placed on a germinating medium in Petri plates and artificially incubated by raising the temperature by 5°C at 5-hourly intervals from 10 to 35°C for 30 h before measuring the three pollen traits. As a result, the *Brassica* cultivars were divided into four groups—heat-tolerant (Kadore, ARC98007, NPZ0591RR, and DSV06200), moderately heat-tolerant (Plainsman, Kronos and DSV05102), moderately heat-susceptible (DSV05101 and KS4085), and heat-susceptible (KS4002, Ceres and KS3077). Thirty-four tomato genotypes were tested under field conditions in a normal (27.1/15.5°C) and summer (39.2/24.4°C) season for heat tolerance, which identified three heat-tolerant genotypes (Pusa Sadabahar, FLA-7171, and NDTVR-60) with high pollen germination and pollen viability, relative to the heat-susceptible genotypes (Floradade and H-86) (Srivastava et al., 2012). In another study, 17 tomato genotypes were evaluated under heat stress (32/26°C) for thermotolerance on the basis of pollen traits (Paupière et al., 2017). The tomato plants were raised in a greenhouse (25/19°C), before being moved to climate chambers when the first flower appeared for the subsequent heat treatment (32/26°C). Thermotolerant genotypes (LA2854, LA1478, and LA0417) had higher pollen viability and pollen numbers than thermosensitive genotypes (LA1719, LA1580, and SWEET4). Similarly, 18 rice (*Oryza sativa*) genotypes varying in heat sensitivity were raised in a greenhouse before being transferred to growth cabinets for high-temperature exposure during anthesis—30°C (control), and 35 and 38°C (heat stress) In this study, two experiments were conducted in two successive years, 1st year experiment involved 30°C (control), and 35°C and 38°C (heat stress) for 2h on the onset of anthesis while 2nd year experiment involved the same set of conditions but heat stress exposure was raised to 6 h (Jagadish et al., 2008). A lower fertility percentage was noticed at 38°C for 6 h compared to 2 h. Genotype N22 had the highest spikelet fertility (86%) and was selected as highly tolerant, while Azucena and Moroberekan had <10% spikelet fertility, thus being the most susceptible genotypes (IR64, CG14); the observations correlated with superior pollen performance at high temperature (Jagadish et al., 2008). *In vitro* pollen germination and pollen tube growth were used to screen 14 cotton cultivars for heat tolerance by raising the temperature by 10°C at 5-hourly intervals from 10 to 50°C for 24 h under controlled environment (Liu et al., 2006). The study revealed that boll retention and boll number per plant were strongly correlated with pollen germination and pollen tube length. The genotypes were categorized into heat-tolerant (Sumian 16 and HLY11), moderately tolerant (JC108, Simian 3, Simian 4, and Lumian 584), moderately susceptible (Zhongmiansuo 12, Zhongmiansuo 41, Zhongmiansuo 9409, Xinyoumian 68, and Sumian 12), and susceptible (TS18, HLY15, and NuCOTN33B).

In legumes, heat stress exposure (47°C) to 44 soybean genotypes identified heat-tolerant (DG 5630RR), heat-intermediate (PI 471938), and heat-sensitive (Stewart III) genotypes based on pollen germination and pollen tube length (Salem et al., 2007). Similarly, heat-tolerant and heat-sensitive mungbean genotypes were identified based on pollen stainability (Suzuki et al., 2001). The plants were exposed to high temperatures (38/28°C) for 24 h in a growth chamber, with pollen stainability recorded on flowers that opened 8–11 days after heat treatment. The heat-tolerant genotype (Haibushi) had higher pollen stainability (60%) than heat-sensitive genotypes (<20%; Kentucky Wonder, Oregon, and Okinawa Local). Heat stress (43/30°C and 45/32°C) in mungbean affected pollen viability, pollen germination, and pollen tube length, more so in the heat-tolerant genotype (SML832) than the heat-sensitive genotype (SML668) in outdoor and controlled conditions (Kaur et al., 2015; Bindumadhava et al., 2018). Exposure of 45 mungbean genotypes to high temperature (42°C) during flowering in the field produced fewer and more shriveled pollen grains, and identified heat-tolerant genotypes (C693357, EC693358, EC693369, Harsha, and ML1299) with superior pollen traits (pollen germination, pollen viability) (Sharma et al., 2016). In chickpea, reproductive traits such as pollen viability, pollen germination, and pollen tube length were used to screen a large number of chickpea genotypes for heat tolerance by delaying sowing to expose plants to temperatures > 32/20°C (day/night); a few tolerant (ICC15614, ICCV92944) and sensitive (ICC10685, ICC5912) genotypes were identified (Kaushal et al., 2013). Another study identified heat-tolerant and heat-sensitive chickpea genotypes using reproductive traits (Devasirvatham et al., 2013) by exposing plants to high temperature (≥35°C). Pollen grains were more sensitive to high temperature than stigmas in both controlled and field conditions. Genotype ICC1205 was identified as heat-tolerant and ICC4567 as heat-sensitive, with a positive correlation between reproductive and yield traits. Lentil is sensitive to heat stress (>35°C), which adversely impairs pollen development and function, resulting in poor pod yields. Based on pollen traits, Kumar et al. (2016) identified heat-tolerant genotypes (FLIP2009-55L, IG2507, and IG4258) after screening 334 lentil accessions for heat tolerance under field conditions (>35/25°C), with a positive correlation between pollen viability and filled pods/plant. In another field study, heat stress (>35/25°C) reduced pollen viability in lentil by up to 78–83% (Sita et al., 2017b), with heat-tolerant genotypes (IG2507, IG3263, IG3745, IG4258, and FLIP2009) maintaining higher pollen germination (48–50%) than heat-sensitive genotypes (28–33%), which was positively correlated with yield. In soybean, exposure of cultivars (i.e., IA3023 and KS4694) and plant introduction lines (PI) lines (i.e., PI393540 and PI588026A) to heat stress (36.5–38.6°C) between gametogenesis and full bloom, as compared to control treatment (29.5–31.6°C; optimum temperature) revealed that the cultivars were more heat tolerant because of greater pollen germination and less distortion in pollen shapes (Djanaguiraman et al., 2019). Combined stress treatment damages the reproductive stages mainly pollen grains to a larger extent (Sehgal et al., 2017). Genetic variations among 38 cotton cultivars for heat and drought were assessed using reproductive and physiological traits. Among reproductive traits, pollen germination as well as pollen viability were tested at two temperature regimes (30 and 38°C) and cumulative heat and drought stress response (CHDSRI) using photosynthetic and reproductive traits was calculated. Based upon CHDSRI, 12 genotypes were categorized as heat and drought sensitive, 20 as intermediate and 6 genotypes as heat and drought tolerant (CT12214, MON11R124B2R2, UA48, MON11R112B2R2, PHY367WRF, and PX53221 1WRF) (Singh K. et al., 2018), which could be potentially used for breeding programs.

## Yield-Based Parameters

Heat stress adversely affects the reproductive and seed-filling stages, leading to severe reductions in crop yield and quality (Sehgal et al., 2018). Various studies have confirmed that the relative performance of plants in terms of yield under heat stress was suitable for selecting genotypes with heat-tolerance mechanisms/traits that can be used for crop improvement. Various traits linked to yield have been used to identify genotypes contrasting for heat tolerance.

Seed formation and seed filling is the last phase in the life cycle of seed plants. Heat stress drastically affects seed development and seed filling in many crop species, which consequently increases the fraction of abnormal and shriveled seeds. Seed development starts from cell division; when seed cells are fully formed, storage reserves start to accumulate in the seed (Egli, 1998). The direct effect of heat stress is reportedly on the division and size of endosperm cells (Commuri and Jones, 1999), such that lower amounts of carbohydrates, proteins, lipids, and starch accumulate in developing seeds. Heat stress also accelerates the rate and duration of seed filling, resulting in more abnormal seeds, which reduces crop yield. Heat stress reduces seed yield by (i) reducing seed number, (ii) reducing seed weight, and (iii) accelerating the seed filling rate (Farooq et al., 2017; Prasad et al., 2017).

### Seed Filling Rate and Duration

Heat stress hastens the seed filling rate and reduces the duration of seed filling. In cowpea, raising the temperature from 15.5 to 26.6°C shortened the seed filling duration by 14–21 days (Nielsen and Hall, 1985). Heat stress impaired the growth of the cotyledons, and reduced the number of endosperm cells and cell expansion in the embryo, which had a negative effect on photosynthate translocation in developing seeds and resulted in shriveled seeds in maize (Jones et al., 1985; Munier-Jolain and Ney, 1998). A heat-stressed environment (>32/20°C) during seed development increased the seed filling rate in six chickpea genotypes, relative to the optimum temperature (Awasthi et al., 2014). The same study revealed that heat stress decreased the duration of seed filling more in heat-sensitive (ICC 4567) than heat-tolerant (ICC1356, ICC15614) genotypes. High temperature (25/20°C) reduced the duration of grain filling by 30% and increased the grain-filling rate by 20% in six wheat genotypes (G1, G2, G3, G4, G5, G6), relative to the control (20/15°C), more so in heat-sensitive (G6) than heat-tolerant (G4) genotypes (Yin et al., 2009).

### Seed Number

Heat stress leads to poor pollination and fertilization, which reduces seed number. In faba bean (*Vicia faba* L), seed number declined with increasing temperature (Bishop et al., 2016). In mungbean, heat-tolerant genotype (SML 832) produced more seeds than heat-sensitive genotype (SML 668) under high temperature (45/32°C) in the field (Kaur et al., 2015). While testing 24 genotypes of common bean in the greenhouse under different temperature regimes (24/21°C, 27/24°C, 30/27°C, 33/30°C), 33/30°C was the most damaging to plants with respect to seed number and seeds/pod, with the reductions more prominent in heat-sensitive genotypes (–66%; A55, Labrador, Majestic, IJR) than heat-tolerant genotypes (–31%; Brio, Carson, G122, HB1880, HT38, Venture) (Rainey and Griffiths, 2005). Heat stress (36/27°C) reduced seed number/pod in 46 of 48 lines of cowpea (*Vigna unguiculata*) evaluated for heat tolerance in a greenhouse; two heat-tolerant lines (B89-600 and TN88-63) did not exhibit reduced seed numbers/pod (Ehlers and Hall, 1998). The average number of seeds/pod varied in the heat-sensitive genotypes (e.g., 3.3 in IT82E-60, 2.9 in Bambey 21 and 3.6 in IT84S-2049), while those of the heat-tolerant genotypes had 6.3 in B89-600 and 8.1 in TN88-63 compared to control values (e.g., 11 in IT84S-2049, 9.6 in IT82E-60, 7.4 in B89-600 and 6.4 in TN88-63).

### Seed Weight

Seed weight represents the ultimate yield of the crop; hence it has been reliably used as a trait to screen for heat tolerance (Sehgal et al., 2018). Chickpea yields declined when genotypes were exposed to various temperature ranges (35/25°C, 40/30°C, and 45/35°C) in a growth chamber, relative to the control (30/20°C) (Kumar et al., 2013). At 40/30°C, the seed weight of heat-sensitive genotypes (ICC14183, ICC5912) declined by 37–45% compared with heat-tolerant genotypes (ICCV07110, ICCV92944). At 45/35°C, heat-tolerant genotypes also experienced a decline in seed weight but heat-sensitive genotypes did not set any pods. Similarly, mungbean genotypes grown outdoors in April, with high temperatures (45/32°C) coinciding with reproductive phase, reduced seed weight by 48.3% in the heat-sensitive genotype (SML668) and 35.1% in the heat-tolerant genotype (SML832), relative to control (Sharma et al., 2016). Likewise, seed weight of lentil grown at high temperature (>32/20°C) in field declined drastically compared to control plants (Bhandari et al., 2016), more so in heat-sensitive genotypes (–50%; LL699 and LL1122) than the heat-tolerant genotype (–33%; LL931). In common bean, heat stress (33/30°C) under field conditions was significant for the selection of heat-tolerant (Brio, Carson, G122, HB1880, HT38, Venture) and heat-sensitive genotypes (A55, Labrador, Majestic, IJR), based on seed weight. At this temperature, seed weight declined by 47% across genotypes, more so in heat-sensitive genotypes (–88%) than heat-tolerant genotypes (–25%) (Rainey and Griffiths, 2005). In cowpea, studies at two locations with varying temperatures (Coachella (41/25°C) and Riverside (36/17°C) assessed the effect of high temperature on the yield of contrasting genotypes (Ismail and Hall, 1999). Yield parameters such as seed weight and seeds/pod reduced drastically, as the temperature increased, however, heat-tolerant genotypes (H36, H8-9, DLS99) at higher temperature (41/25°C) retained more seed weight (193 mg/seed) than heat-sensitive genotypes (CB5, CB3, DLS127), which had smaller seeds with an average weight of 168 mg. Screening experiments on Pearl millet, conducted over a period of 3–4 years (2009–2012) at ICRISAT, India, involving 221 hybrid parental lines (both B- and R-lines), 53 germplasm accessions and 4 improved populations over 4-year period showed large genetic variability in seed set at daily maximum air-temperature of ≥ 42 °C during flowering. Five hybrid seed parents (ICMB 92777, ICMB 05666, ICMB 00333, ICMB 02333, and ICMB 03555) and a germplasm accession IP 19877 with 61–69% seed set as compared to 71% seed set in a heat tolerant commercial hybrid 9444 (used as a control) was identified. A comparative study on 23 hybrids and their parents for seed set at high air temperature (>42°C) showed heat tolerance as a dominant trait, indicating that heat tolerance in one parent would be ample to generate heat tolerant hybrids in pearl millet (Gupta et al., 2015). In sub-Saharan Africa, 24 elite durum wheat breeding lines and cultivars were tested for adaptation to warm environments at two stations: Kaedi, Mauritania and Fanaye, Senegal. Top grain yield was recorded at 5,330 kg ha^–1^ and the average yield at 2,484 kg ha^–1^. Biomass and spike fertility (i.e., number of seeds produced per spike) were found to be the most vital adaptive traits to warm environments. The study showed three genotypes (“Bani Suef 5,” “DAWRyT118,” and “DAWRyT123”) as the most stable and high yielding; while the last two genotypes were the best performers (Sall et al., 2018).

Combined drought and heat stress were found to be greatly detrimental for production potential of crops. Thus, lentil genotypes were evaluated for their response to impacts of combined drought and heat stress (drought tolerant: DPL53 and drought sensitive: LL699) (Sehgal et al., 2019). The heat and drought (33/28°C with 50% field capacity) treatments were imposed to determine to effects on yield traits (seed filling duration, seed filling rate, seed number/plant, and seed weight/plant). Under combined stress, a decline in seed filling duration by 5.4–8.9 days, seed growth rate by 44–60.2%, seed number/plant by 35–48.7%, seed weight/plant by 47–59% compared to control. This reduction pattern was more drastic in heat sensitive genotype than heat tolerant genotype. A field experiment on 300 maize inbred lines test-crossed to CML539 was conducted at multiple locations (Tlaltizapán, México (18°41/c N, 99°07/c W, and 940 m asl), Kiboko, Kenya (2°21/c S, 37°72/c E, and 975 m asl), Chiredzi, Zimbabwe (21°01/c S, 31°34/c E, and 430 m asl), at the Nakhonsawan Field Crops Research Center in Takfa, Thailand (15°21/c N, 100°30/c E, and 87 m asl), and at the ICRISAT experimental station in Hyderabad, India) to evaluate their response to reproductive stage drought stress, heat stress, and combined drought and heat stress. The study identified few lines (notably La posta Sequia C7-F64-2-6-2-2 and DTpYC9-F46-1-2-1-2) having higher tolerance to drought and combined drought and heat stress. The findings indicated that tolerance to individual stresses was genetically distinct from tolerance to combined stresses. The assessment indicated that most of the current drought donors and key inbreds used in widely grown African hybrids were sensitive combined drought and heat stresses. The identified lines, as mentioned above, need to be introduced into breeding programs for maize (Cairns et al., 2013).

## Breeding for Heat Tolerance Involving Contrasting Genotypes

Breeding techniques remain one of the inexpensive and viable approaches for developing heat stress tolerance in crop plants (Priya et al., 2018). Field-based screening of crop gene pool and landraces for yield and heat stress tolerance in targeted environments is a way to develop heat tolerant genotypes in various crop plants (Craufurd et al., 1998; Hede et al., 1999; Ntare et al., 2001; Jagadish et al., 2008; Scafaro et al., 2010; Krishnamurthy et al., 2011; Dhanda and Munjal, 2012; Pradhan et al., 2012). The breeders also focus toward yield and yield-related traits under heat stress so that genotypes/progeny lines with higher yield under heat stress can be selected. Varieties possessing heat stress tolerance as well as higher yields will ensure adequate food to the world’s burgeoning population under global warming. To develop heat tolerant crop varieties, contrasting donor parents are crossed, progenies advanced using various crop breeding strategies and desirable heat tolerant segregants are selected. Finally, heat tolerant homozygous lines are evaluated for yield and other useful agronomic traits under appropriate environments followed by possible release as a variety/ies. For transfer of heat tolerance to high yielding but heat sensitive mega crop varieties (varieties that occupy large area) from heat tolerant landraces or wild relatives, backcross breeding with recurrent parent remains an effective strategy as it allows for the recovery of the genome of recurrent parent, thereby traits of mega variety, with an addition of heat tolerance. To broaden the genetic base for heat tolerance, next generation breeding schemes viz., development of Multiparents Advanced Generation Intercross (MAGIC) and Nested Association Mapping (NAM) population are also receiving wider attention (Li H. et al., 2018).

Morpho-physiological and phenological traits could play an important role in contributing toward heat stress adaptation as these could act as surrogate traits for selecting heat tolerance (Reynolds et al., 2007). These physiological traits range from early phenology (Gaur et al., 2015), canopy temperature (Kumar et al., 2012; Mondal et al., 2013), chlorophyll fluorescence, chlorophyll content (Ristic et al., 2007; Kumar et al., 2013), cell membrane stability (Blum and Ebercon, 1981), stay green trait or delayed senescence (Thomas and Howarth, 2000; Ristic et al., 2007), pollen and pollen related traits (Devasirvatham et al., 2010; Kaushal et al., 2013; Djanaguiraman et al., 2018, Djanaguiraman et al., 2019) to water soluble carbohydrates in stem (Schittenhelm et al., 2020). The physiological trait-breeding has gained great attention for improving plant adaptation to heat stress in various crop plants especially in wheat (Reynolds et al., 2007; Reynolds and Langridge, 2016). A focus on selection of physiological traits that are correlated with yield either directly or indirectly could increase chances of accumulation of yield contributing genes thereby ensuring higher plant yield under heat stress (Reynolds and Langridge, 2016). In developing heat tolerance in wheat, the cross-species gene transfer system was used wherein three heat-tolerant accessions of *Aegilops tauschii* (wild genotype) were crossed with bread wheat (*Triticum aestivum* L.) cultivar “PBW 550” (Sehgal et al., 2011). The BC_1_F_4_ lines derived from these crosses that possessed improved cell membrane stability, TTC and chlorophyll retention under heat stress were selected (Sehgal et al., 2011). For winter sown crops, early phenology allows plants to escape heat stress (Bueckert et al., 2015). For such crops, selection for earliness could be an important option to develop crop varieties that escape heat stress thereby escaping the damage caused by heat. As reproductive processes are most vulnerable to heat stress, physiological screening of genotypes for two reproductive traits, i.e., better pollen viability and pollen germination under heat stress could lead to the identification of heat tolerant genotypes as stability of these two traits under heat stress will ensure better fertilization, adequate seed set and improved grain yield (Devasirvatham et al., 2013; Poli et al., 2013). Relying on higher pollen germination and better seed setting capability Nguyen et al. (2013) identified two sorghum R9403463–2-1 and IS8525 genotypes from a set of diverse sorghum genotypes originated from United States, Australia, Africa and Asia. Likewise, several promising genotypes viz., PI609489, AQL33/QL36; CCH2; IS 8525 (Singh V. et al., 2015) due to their better seed setting ability and Macia, BTx378, SC155 (Sunoj et al., 2017) having better pollen germination capability and maintaining high grain yield under heat stress were identified. Given the field screening of large set of germplasm and hybrid parental lines of Pearl millet under high temperature stress, a wide range of genetic variability for seed setting was noted in under high temperature stress (Gupta et al., 2015). Several parental lines viz., ICMB 92777, ICMB 05666, ICMB 00333 along with IP 19877 germplasm accession exhibited better seed setting under heat stress and thus could be used in developing heat tolerant hybrid Pearl millet (Gupta et al., 2015). Likewise, Jukanti et al. (2017) underscored the importance of CZH 233, CZP 9603, CZI 2011/5, and CZMS 21A genotypes due to their better seed setting higher capability of grain yield for developing superior Pearl millet genotypes under heat stress. Likewise, the potentiality of “Norchip” and “Désirée” potato cultivars in potato breeding program for improving genetic gain because of their better photo-assimilate transport from leaf to tuber under heat stress has been discussed (Basu and Minhas, 1991; Ahn et al., 2004).

Heat stress tolerance is a polygenic trait. Classical genetics was earlier used to identify the genetic bases of heat tolerance in various field and vegetable crops (Patel and Hall, 1988; Marfo and Hall, 1992; Gupta et al., 2015; Jha et al., 2019), this approach, however, could not completely explain the genetic nature of heat stress tolerance because of its multigenic nature (Upadhyaya et al., 2011). Subsequent advances in molecular marker technology has allowed identification and precise mapping of genes/QTLs governing heat stress tolerance several crops such as rice (Gui-lian et al., 2009; Lei et al., 2013; Wei et al., 2013; Li M. et al., 2018), maize (Inghelandt et al., 2019), wheat (Mason et al., 2010; Pinto et al., 2010; Paliwal et al., 2012; Lopes-Caitar et al., 2013; Sharma et al., 2017), chickpea (Paul et al., 2018), cowpea (Pottorff et al., 2014), *Brassica* (Branham et al., 2017) and tomato (Wen et al., 2019). Marker assisted selection can be used to transfer heat tolerant QTLs/genomic region to the elite but heat stress sensitive genotypes if genetic maps with sufficient marker density are available (see Jha et al., 2014). The approach has been successfully employed in rice (Ye et al., 2012; Shirasawa et al., 2013), wheat (Pinto et al., 2010; Bennett et al., 2012; Bonneau et al., 2013) and tomato (Grilli et al., 2007) to transfer QTLs governing heat tolerance. Considering potato, Trapero-Mozos et al. (2017) discussed the scope of introgression of *HSc70* allelic variant contributing toward enhancing yield under heat stress into high yielding potato cultivars through marker assisted breeding for improving heat tolerance in potato. Advent of improved sequencing technologies that allow faster sequencing of genomes at lower costs led to generation of profuse SNP markers that enabled genome-wide association studies (GWAS) for elucidating novel genomic regions controlling heat stress tolerance. GWAS for identifying heat stress tolerance genomic regions have been conducted in rice (Lafarge et al., 2017), maize (Yuan et al., 2019), wheat (Maulana et al., 2018), barley (Cantalapiedra et al., 2017), pea (Tafesse et al., 2020), chickpea (Thudi et al., 2014; Jha et al., 2018; Varshney et al., 2019), and in *Brassica* (Rahaman et al., 2018).

## Transcriptomics

Previously cDNA-AFLP and microarrays were employed for identifying heat tolerance genes in various crop plants (Bita et al., 2011; Johnson et al., 2014). After the advent of crop-specific gene chips, microarrays became the method of choice for estimating changes in gene expression upon exposure to abiotic stress e.g., Gene Chip wheat genome array in wheat (Qin et al., 2008), Affymetrix GeneChip^®^ Tomato Genome Array in tomato (Frank et al., 2009), Affymetrix 22K Barley 1 Gene Chip microarray in barley (Mangelsen et al., 2011) and *Brassica* 95k EST microarray in *Brassica* (Yu et al., 2014). Microarray-based analysis by Johnson et al. (2014) provided insights into various genes involved in heat tolerance in sorghum. Major revolution in our understanding of genes involved in heat stress tolerance occurred after the advent of modern DNA sequencing technologies that allowed sequencing of whole transcriptomes, a technique called transcriptomics/transcriptome sequencing/whole genome transcriptome sequencing/whole genome expression profiling. Transcriptomics allowed identification of various heat tolerant candidate genes with greater precision in rice (González-Schain et al., 2016; Mangrauthia et al., 2016; Fang et al., 2018), wheat (Liu et al., 2015), maize (Shi et al., 2017), chickpea (Agarwal et al., 2016), and soybean (Gillman et al., 2019). Transcriptome analysis of contrasting heat tolerant and sensitive lines led to identification of 35 differentially expressed transcripts between the contrasting rice lines, 21 of which were functionally validated (Liao et al., 2015). The study suggested involvement in oxidation-reduction, metabolic activity, defense response and photosynthesis activity in heat tolerance (Liao et al., 2015). Zhao et al. (2018) explored several *Hsp20* family genes involved in heat stress response across the whole genome in potato. A total of 14 *Hsp20* genes displaying up-regulatory role under heat stress in potato was confirmed through real-time quantitative PCR. RNA-seq analysis of maize seedling treated with heat stress unveiled myriads of up and down regulated genes related to photosynthesis, protein synthesis and biosynthesis of various metabolites including zeatin, brassinosteroids (Frey et al., 2015; Shi et al., 2017). Further, Zhao et al. (2019) unearthed the involvement of 5,400 non-additive genes specific to heat stress through transcriptome analysis of parental lines and F_1_ hybrid maize seedlings under heat stress conditions. RNA-seq technology not only identified the genes for heat tolerance but also the non-coding RNAs that were involved in regulating heat stress responses in various crops (Wang et al., 2011; Xin et al., 2011; Yu et al., 2013; Mangrauthia et al., 2017).

## Proteomics

Gene expression enhanced our understanding of mechanisms of heat stress tolerance significantly, however, gene transcripts do not directly influence plants’ responses to stresses. Instead the proteins/enzymes, the gene products, modify plants’ metabolite pool in response to external stimulus. To understand better, the mechanisms of stress tolerance, studies of the proteome, i.e., entire set of proteins in a cell or organ were initiated. Prior to proteomics, proteins suspected to play role in heat tolerance were analyzed by MALDI TOF MS/MS analysis, e.g., rice (Han et al., 2009; Jagadish et al., 2010; Liao et al., 2014). Further advances in proteomics strengthen our understanding of identification of the proteins that confer thermotolerance in plants. Proteomics analysis of two contrasting rice genotypes, N22 (tolerant) and Gharib (sensitive), showed that heat tolerance of N22 was due to higher capability of mediating renaturation of stress damaged proteins, higher efficiency in repairing ribosomal protein, higher upregulation of proteins involved in calcium signaling and phytohormone synthesis and protein modifications under high night temperature at early grain filling stage (Shi et al., 2013). The functional role of proteins that contribute to heat tolerance ranges from oxidation-reduction, cellular metabolic activity to defense responses (Lu et al., 2017; Zhang et al., 2017). In this context, Zhang et al. (2017) identified various proteins by analysis of grains of contrasting heat tolerant rice lines by employing isobaric tags for relative and absolute quantitation (iTRAQ) methods (Zhang et al., 2017). Similarly, by employing iTRAQ technique, Lu et al. (2017) identified 258 heat responsive proteins from wheat leaf, most of which were involved in chlorophyll synthesis, carbon fixation and redox regulation under heat stress. Various proteins such as HSP, those related to anti-oxidant mechanism, and glycolysis were involved in adaption of grape to heat stress as revealed through iTRAQ analysis (Liu et al., 2014). Proteomics analysis of ethylene pre-treated tomato pollen by LC-MS/MS suggested that various proteins help in protecting pollen development and function through higher abundance of protein synthesis and upregulating stress protecting proteins that maintain cellular redox state under heat stress (Jegadeesan et al., 2018). Proteomics analysis by 2-DE technique allowed identification of important heat shock proteins viz., HSP26, HSP16.9, and unknown HSP/Chaperonin contributing to heat stress tolerance in maize (Abou-Deif et al., 2019). Considering contributory role of proteins adapting roots under heat stress, Valdes-Lopez et al. (2016) reported the involvement of both up and down regulatory proteins contributing to heat tolerance in soybean root. Recently, proteomics analysis deduced that protein phosphorylation and protein acetylation could regulate heat tolerance by modulating photosynthesis protein in grape (Liu et al., 2019). The proteins involved in heat tolerance elucidated through proteomics analysis could serve as biomarkers for identifying heat tolerant cultivars in various crop plants. Participatory role of miR156 targeting SPL transcription factor in *A. thaliana* (Stief et al., 2014), miRl60, miRl66, and miRl67 in wheat and barley (Xin et al., 2010), IbmiR397 targeting *laccase gene* in sweet potato (Yu et al., 2020) controlling heat stress response are worth mentioning.

## Metabolomics

Metabolomics, the study of metabolites in a cell or organ, enhance our understanding of novel metabolites that contribute to plant adaptation to heat stress (Bokszczanin and Fragkostefanakis, 2013). Metabolomics have unraveled the key metabolites ranging from sugars, proteins and lipids participating in key biological processes to anti-oxidants and defense molecules in response to heat stress (Li T. et al., 2015; Chebrolu et al., 2016; Muhlemann et al., 2018; Salvi et al., 2018). Metabolomics at specific plant stages viz., seed germination, vegetative, reproductive, grain formation and grain filling have broadened our understanding of metabolites involved in heat stress responses at different development stages (Wang et al., 2015; Mangrauthia et al., 2016; Spicher et al., 2016; Templer et al., 2017; Muhlemann et al., 2018; Qu et al., 2018; Thomason et al., 2018). Metabolomics provided novel insights into the role of various lipids viz., plastidic glycerolipids, oxidized glycerolipids in regulating heat stress responses in wheat leaves (Narayanan et al., 2016), that of α-tocopherol and plastoquinone in maintaining the photosynthesis apparatus in tomato under heat stress (Spicher et al., 2016) and that of galactinol in minimizing excessive ROS activity in chickpea under heat stress (Salvi et al., 2018). Metabolomics also emphasized the role of sugars in anthers such as glucose−6−P, fructose−6−P, glucose, maltose and *myo*−inositol in improving heat stress acclimation in N22 (heat-tolerant) rice genotype (Li X. et al., 2015). Likewise, the ameliorative role of various anti-oxidant phenolic compounds viz., flavonoids, flavonols, tocopherols in heat tolerance by preventing ROS mediated negative effect on pollen tube germination in tomato (Muhlemann et al., 2018) and also during seed development in soybean (Chebrolu et al., 2016) are other examples of the use of metabolomics in improving knowledge of heat stress tolerance mechanisms. At post anthesis stage, metabolites viz., drummondol, anthranilate appear to regulate heat stress response in wheat flag leaves (Thomason et al., 2018). The studies pinpoint that metabolomics along with system biology approaches could significantly enhance significantly our understanding of various metabolites produced in response to heat stress (Janni et al., 2020) and would be a vital tool to develop heat tolerant crops in agriculture.

## Conclusion and Future Perspectives

The past few decades have seen considerable developments in genetics, biochemical, genomics, transcriptomics, proteomics and metabolomics approaches to enhance the understanding of heat stress tolerance. However, basal thermotolerance remains the major tool to develop agronomically superior heat tolerant cultivars for agricultural crops. Basal thermotolerance is primarily evaluated by exposing small or large sets of germplasm (accessions, cultivars, wild relatives) under controlled (laboratory, screen/greenhouse) or natural field environments to stressful temperatures. These tests have identified several sources of heat tolerance in various crop gene pools and landraces, which may act as potential candidates/donors of heat stress tolerance for developing heat tolerant cultivars using conventional or modern breeding approaches (Table 1). In some instances, heat tolerant genotypes have been directly released as cultivars (as in Chickpea) owing to their agronomic superiority. In addition to heat stress tolerance, contrasting genotypes are also being evaluated for diverse traits related to phenology, growth, physiology and biochemistry, genes, and reproductive biology. Of the several traits being evaluated for heat stress tolerance in crops, the majority of studies have indicated pollen function to be highly sensitive to heat stress, thus making it one of the vital selection traits for heat tolerance. Evaluation of thousands of germplasm or progeny lines for several traits associated with heat tolerance in a short span of time is needed to fasten the breeding for heat tolerance. High-throughput phenotyping that allows choosing important traits as selection criteria for heat tolerance can facilitate identification of genotypes for heat stress tolerance as well as other desirable agronomic traits in a short span of time but high throughput phenotyping requires high investment and is available with only a few laboratories around the world. In addition to it, remote sensing tools (UAVs with spectral and thermal imaging camera) can be effectively deployed under realistic field environments to screen thousands of germplasm or progeny lines.

**TABLE 1:** Few selective heat-tolerant genotypes identified for various crops involving various traits (details in the text).

**Crop**	**Traits used**	**Screening method**	**Promising heat-tolerant genotypes**	**Country**	**References**
**1. Cereals**					
**Barley** (*Hordeum vulgare L.*)	Stay green trait	Field experiments (> 40°C)	L6 and L8 and L3 and L10	Iran	Bavei et al., 2011
	Chlorophyll fluorescence	Growth chamber (45°C)	Ig, Im, and Tz	North Africa	Oukarroum et al., 2016
**Wheat** (*Tritium aestivum L*.)	Stay green trait	Field experiments (32°C)	CB367(BB#2/PT//CC/INIA/3/ALD“S,” CB = 333(WL711/3/KAL/BB//ALD“S”and CB335(WL711/CROW“S”//ALD#1/CMH7 7A.917/3/HI666PVN“S”)	Pakistan	Rehman et al., 2009
	Canopy temperature depression	Field experiment (41°C)	HD 2932, HD 2864, HD 3095, HI 8703, HUW 234	India	Saxena et al., 2016
**Rice** (*Oryza sativa* L.)	Pollen-based	Growth cabinets (35°C, 38°C)	N22	United Kingdom	Jagadish et al., 2008
	Cell membrane thermostability	Phytotron (40°C)	F473	America	Sanchez-Reinoso et al., 2014
**Maize** (*Zea mays* L.)	Plant height	Field experiments (>40°C)	DTPYC9F119	India	Debnath et al., 2016
	Root system architecture	Growth chamber (37°C)	H16, CML444, SC-Malavi	United States	Trachsel et al., 2010
**2. Legumes**					
**Chickpea** (*Cicer arietinum*)	Photosynthetic rate	Field environment (25 to 40°C)	Pusa 1103, Pusa 1003, KWR 108, BGM 408, BG 240, PG 95333, JG 14, BG	India	Kumar et al., 2017
	Cell membrane thermostability	Growth chamber (40–45°C)	ICCV07110, ICCV92944, ICC1205	India	Kumar et al., 2013
**Lentil** (*Lens culinaris* Medik.)	Biomass Stomatal conductance Chlorophyll fluorescence Chlorophyll content Sucrose Oxidative stress and antioxidants	Field study (>32/20°C)	IG2507, IG3263, IG3745, IG4258, and FLIP2009	India	Sita et al., 2017a
	Cell membrane thermostability	Growth chamber (34°C)	FLIP2009, Ranjan, Moitree, 14-4-1, IC201710, IC208329	India	Choudhury et al., 2012
**Mungbean** (*Vigna radiata* L.)	Pollen-based Biomass Chlorophyll fluorescence Oxidative stress and antioxidants	Field experiments (>40/28°C)	EC693357, EC693358, EC693369, Harsha, and ML 1299	India	Sharma et al., 2016
**Common bean** (*Phaseolus vulgaris* L.),	Chlorophyll fluorescence	Growth chamber (42°C)	*Ranit* and *Nerine RS*	Bulgaria	Petkova et al., 2007
	Seed weight Seed number	Field conditions (27/24°C, 30/27°C, and 33/30°C)	Brio, Carson, G122, HB1880, HT38, Venture	Switzerland	Rainey and Griffiths, 2005
**Alfalfa** (*Medicago sativa*)	Biomass Chlorophyll fluorescence Cell membrane thermostability	Greenhouse and growth incubators (38/35°C)	Bara310SC	China	Wassie et al., 2019
**Soybeans** (*Glycine max* L. Merr.)	Photosynthetic rate	Field experiments (36.5–38.6°C)	IA3023 and KS4694	United States	Djanaguiraman et al., 2019
	Metabolites	Lab experiments (36°C/24°C; 46/26°C)	PI587982A	South America	Chebrolu et al., 2016
**Cowpea** (*Vigna unguiculate* L. Walp)	Seed weight	Field studies (41/25°C)	H36, H8-9, DLS99	United States	Ismail and Hall, 1999
	Seed number	Greenhouse conditions, (36/27°C)	B89-600 and TN88-63	United States	Ehlers and Hall, 1998
**3. Oil seed crops**					
**Indian Mustard** (*Brassica juncea* L)	Plant height	Field conditions (34°C)	BPR-538-10, NRCDR-2, RH-0216	India	Chauhan et al., 2009
**Canola** (*Brassica napus*)	Pollen-based	Field studies (10–35°C)	Kadore, ARC98007, NPZ0591RR, and DSV06200	United States	Singh et al., 2008
	Root system architecture	Growth chamber (32°C)	Invigor 5440	Canada	Wu et al., 2017
**Peanut** (*Arachis hypogaea* L.),	Carbon isotope discrimination	Polytunnels (40/28°C)	Spanish botanical type	United Kingdom	Craufurd et al., 1999
**Peanut** (*Arachis hypogea*)	Heat shock proteins	Controlled environment (50°C)	ICGS 76, COC038, COC068, COC050, COC041	United States	Selvaraj et al., 2011
**Cotton** (*Gossypium hirsutum* L.)	Cell membrane thermostability	Field conditions (>44°C)	NIA-80, NIA-81, NIA-83, NIA-84, NIA-M-30, NIA-M31, NIA-HM-48, NIA-HM-327, NIA-H-32, NIA-HM-2-1, NIA-Bt1, NIA-Bt2, NIA-Perkh, CRIS-342, CRIS-134, NIAB-111 and check variety Sadori	Pakistan	Abro et al., 2015
	Chlorophyll fluorescence	Green house (30 and 40°C)	DP393, VH260 and DP 210 B2RF	Africa	Van der Westhuizen et al., 2020
**4. Vegetable crops**					
**Potato** (*Solanum tuberosum* L.)	Heat shock proteins	Growth chamber (40°C)	Norchip, Desiree	Baltimore	Ahn et al., 2004
**Tomato** (*Solanum lycopersicum* L.)	Pollen-based	Field conditions (39.24/24.42°C)	Pusa Sadabahar, FLA-7171, NDTVR-60	Japan	Srivastava et al., 2012
	Sucrose	Growth chambers (31/25°C) or Greenhouses (32/26°C)	FLA 7516, Hazera 3018, Hazera 3042, and Saladate	Israel/United States	Firon et al., 2006
**Cabbage** (*Brassica* species)	Metabolites	Control environment (25–35°C)	Yoshin, Kenshin and full white	Japan	Hossain et al., 1995
**Cucumber** (*Cucumis sativus* L.)	Cell membrane thermostability	Growth room (40/32°C)	L-3466, Desi Cucumber	Pakistan	Ali et al., 2019

Plant heat tolerance being a quantitative trait is highly influenced by G × E interactions and genetic inheritance of heat tolerance remains challenging. Large scale DNA-based marker development during the last decade led to mapping of QTLs linked to heat tolerance in various crops (Jha et al., 2014; Janni et al., 2020). Advances in sequencing technologies especially, next generation sequencing (NGS), genotyping by sequencing (GBS), and other high throughput genotyping platforms have facilitated narrowing down of the heat tolerance QTL regions for analysis of candidate genes (Xu et al., 2017; Kilasi et al., 2018; Inghelandt et al., 2019; Tadesse et al., 2019). Given the huge number of novel SNPs developed recently and GWAS in large set of global crop germplasm, it became possible to identify novel haplotypes/genomic regions controlling heat tolerance (Paul et al., 2018; Varshney et al., 2019; Khan et al., 2020; Weckwerth et al., 2020) and allowed for the assessment of genetic diversity at nucleotide-scale. High throughput phenotyping coupled with advanced imaging devices, unmanned vehicles and machine learning, deep learning approaches and molecular genetics tools can further enhance the accuracy of selection of genomic regions associated with heat tolerance. The developments in marker and sequencing technologies are expected to allow genome wide marker profiling facilitating genomic selection for heat tolerance (Tricker et al., 2018; Inghelandt et al., 2019) and thus, rapid breeding for the development of varieties with novel genetic combinations. Similarly, advances in proteomics, transcriptomics and metabolomics will further unravel the complexity of heat stress tolerance in crops by identifying missing links in the current information. A combination of these approaches could allow for the quantifying of plant heat stress responses, spatially and temporally, at a large scale, thus narrowing the “genotype-phenotype gap” (Fahlgren et al., 2015; Singh A. et al., 2015; Singh A. K. et al., 2018; Pinto et al., 2016). Corresponding to breeding approaches, current developments in the spatial and temporal expressions of engineered genes or pathway engineering by the targeted editing of genomes using CRISPR–Cas technology can be used for development of heat tolerant designer crops. A better knowledge of plant cellular mechanisms associated with heat tolerance and increased yields would be vital to drive essential gains in crop improvement, which can be greatly assisted by exploring the genetic diversity in heat tolerance, and put into practice by genome-scale breeding, precisely done gene engineering and better agronomic management practices.

## Author Contributions

HN conceived the outline. All authors contributed in preparing various sections of this manuscript.

## Conflict of Interest

The authors declare that the research was conducted in the absence of any commercial or financial relationships that could be construed as a potential conflict of interest. The reviewer PK declared a past co-authorship with one of the authors KS to the handling editor.
